# Polyploidy, EZH2 upregulation, and transformation in cytomegalovirus-infected human ovarian epithelial cells

**DOI:** 10.1038/s41388-023-02813-4

**Published:** 2023-08-26

**Authors:** Ranim El Baba, Sandy Haidar Ahmad, Franck Monnien, Racha Mansar, Frédéric Bibeau, Georges Herbein

**Affiliations:** 1 Department of Pathogens & Inflammation-EPILAB Laboratory EA4266, University of Franche-Comté, Besançon, France; 2 Department of Pathology, CHU Besançon, Besançon, France; 3 Department of Virology, CHU Besançon, Besançon, France

**Keywords:** Cancer, Microbiology

## Abstract

Human cytomegalovirus (HCMV) infection has been implicated in epithelial ovarian cancer (OC). Polyploidy giant cancer cells (PGCCs) have been observed in high-grade serous ovarian carcinoma (HGSOC); they possess cancer stem cell-like characteristics and give rise to progeny cells expressing epithelial-mesenchymal transition (EMT) markers. EZH2 plays a potential oncogenic role, correlating with high proliferative index and tumor grade in OC. Herein, we present the experimental evidence for HCMV as a reprogramming vector that elicited human ovarian epithelial cells (OECs) transformation leading to the generation of “*CMV-transformed Ovarian cells*” (CTO). The infection with the two high-risk clinical strains, namely HCMV-DB and BL provoked a distinct cellular and molecular mechanisms in infected OECs. EZH2 upregulation and cellular proliferation were curtailed by using EZH2 inhibitors. The HGSOC biopsies were characterized by an elevated EZH2 expression, possessing a strong positive correlation between the aforementioned marker and HCMV. From HGSOC biopsies, we isolated three HCMV clinical strains that transformed OECs generating CTO cells which displayed proliferative potentials in addition to EZH2 upregulation and PGCCs generation; these features were reduced upon EZH2 inhibition. High-risk HCMV strains transformed OECs confirming an HCMV-induced epithelial ovarian cancer model and highlighting EZH2 tumorigenic properties. Our findings might be highly relevant in the pathophysiology of ovarian tumors thereby nominating new targeted therapeutics.

## Introduction

Epithelial ovarian cancer (OC), the most common and life-threatening cancer amongst gynecologic malignancies, has a 5-year age-standardized survival rate of 30–40%. Nearly 75% of all OC cases are diagnosed at late stages [1]. Despite the advances in treatment modalities, the overall survival remains low for stage III and stage IV accounting for 40 and 20%, respectively. Optimal surgery and platinum-based chemotherapy (such as carboplatin and cisplatin) remain the mainstay of treatment often combined with paclitaxel, gemcitabine, a humanized monoclonal antibody targeting vascular endothelial growth factor (VEGF) bevacizumab, pegylated liposomal doxorubicin, cyclophosphamide, topotecan, poly-ADP-ribose- polymerase (PARP) inhibitors, and immune check point inhibitors especially for recurrent OC [2–4]. In some cases, due to the poor blood supply to the peritoneal surface, hyperthermic intraperitoneal chemotherapy (HIPEC) has been used as a therapeutic alternative [5].

Human cytomegalovirus (HCMV) is a herpesvirus that infects between 40 and 95% of the worldwide population [6]. In healthy individuals, primary HCMV infection is asymptomatic. HCMV establishes a life-long chronic latency primarily in monocytes, tissue macrophages, and myeloid lineage CD34^+^ hematopoietic progenitor cells, with periodic reactivation [7]. HCMV genomic variability could contribute to its oncomodulatory role where HCMV will encourage the development and spread of tumor cells [8, 9]. Lately, an oncogenic role of HCMV has been underlined where the high-risk HCMV strains directly transform primary cells [10].

Polyploidy, a condition in which a normally diploid cell acquires additional sets of chromosomes, is formed via cell-cell fusion or endoreplication [11]. Polyploidy giant cancer cells (PGCCs) have been confirmed to possess cancer stem cell (CSC)-like characteristics, and give rise to progeny cells through asymmetric division which express epithelial-mesenchymal transition (EMT)-related markers thereby promoting invasion and migration [12–14]. Studies have shown that PGCCs generation was induced by anti-cancer therapies where the latter convert proliferating tumor into dormant cells [15]. The presence of PGCCs in OC has been preferentially reported in poor prognosis cancers participating in tumor relapse and therapy resistance [16]. Moreover, previous studies revealed the detection of PGCCs harboring HCMV; a significant correlation was shown between PGCCs presence and HCMV in breast cancer biopsies [17–20]. Besides PGCCs, tetraploidy has been reported in approximately 26% of solid tumors [21]; it’s usually formed through cell fusion, endoreduplication, mitotic slippage, or cytokinetic failure, the latter two being the main mechanisms [21, 22]. It has been reported that tetraploid cells induce aneuploidy and transformation of mouse ovarian surface epithelial cells [23]. Aneuploidy post-tetraploidization is observed mainly in p53-inactivated cells [21]. p53 and telomerase depletion generates a state of chronic DNA damage, therefore, resulting in tetraploidization [21, 24].

Classical OC risk factors including age, family history, epigenetic as well as genetic alterations might generate a tumor-supporting cellular environment, where some oncogenic viruses reside and advance their oncogenic potential [1]. EZH2 dysregulation was associated with the development, progression, and therapeutic resistance of many tumors, particularly OC [25]. Several studies showed that EZH2 overexpression is associated with high-grade serous ovarian carcinoma (HGSOC) [25, 26]. Another study revealed that invading PGCCs possess strong EZH2 expression [27]. All in all, EZH2 potentially serves as an effective therapeutic target [25]. Moreover, there exists a strong emerging evidence showing HCMV infection prevalence in breast, cervical, colon, liver, prostate, and brain cancer patients [28–30]. HCMV-IE and pp65 proteins were detected in 82 and 97% of epithelial ovarian cancer, respectively [31]. Prominent HCMV-IE protein expression was linked to a higher stage of OC [31]. Further, CMV DNA was detected in 70% of cancerous ovarian tissues and was significantly higher compared to benign tumor cases [32]. Recent studies have identified high HCMV expression in OC biopsies that were associated with poor survival outcomes [33].

To investigate the potential role of HCMV infection in epithelial ovarian cancer, especially in HGSOC, human ovarian epithelial cells (OECs) were infected with low (KM and FS) and high-risk (DB and BL) oncogenic HCMV clinical strains; the latter previously transformed human astrocytes and human mammary epithelial cell [17, 30, 34]. In this study, we assessed the transforming potential of the two high-risk clinical strains, HCMV-DB and HCMV-BL, and investigated their molecular and cellular features that appeared in the long-term “*CMV-transformed Ovarian cells*” (CTO) cultures. Additionally, from HGSOC biopsies that revealed high EZH2 expression, we isolated three clinical HCMV strains that displayed oncogenic and stemness features when cultivated on OECs with enhanced EZH2 expression that could be curtailed by EZH2 inhibitors.

## Results

### OECs chronically infected with HCMV-DB and BL strains generated CTO cells with PGCCs

**Fig. 1:**
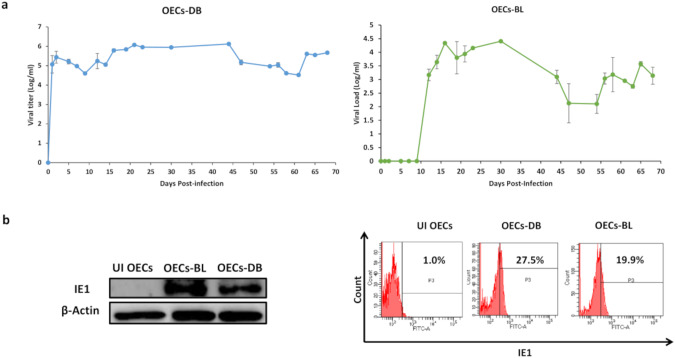
Replication of high-risk HCMV strains in OECs cultures. **a** Time-course of the viral titer in the supernatant of OECs infected with HCMV-DB and BL as measured by IE1-qPCR. **b** Immunoblotting data of IE1 in uninfected OECs lysates and OECs infected with HCMV-DB and BL (day 5 post-infection). β-actin was used as loading control. IE1 expression by FACS in acutely infected OECs-DB and BL; UI OECs were used as a control.

Upon studying the tropism exhibited by HCMV in OECs, all HCMV clinical strains replicated showing a peak viral replication at day 21, 16, 16, and 19 post-infection in OECs infected with HCVM-DB, HCMV-BL, HCMV-KM, and HCMV-FS, respectively (Fig. 1a and Supplementary Fig. 1a). The peak level of HCMV productive infection was 6 and 4 logs in OECs-DB and BL, respectively (Fig. 1a). HCMV-IE1 and pp65 proteins were detected in OECs-DB and BL (Fig. 1b and Supplementary Fig. 2).

**Fig. 2:**
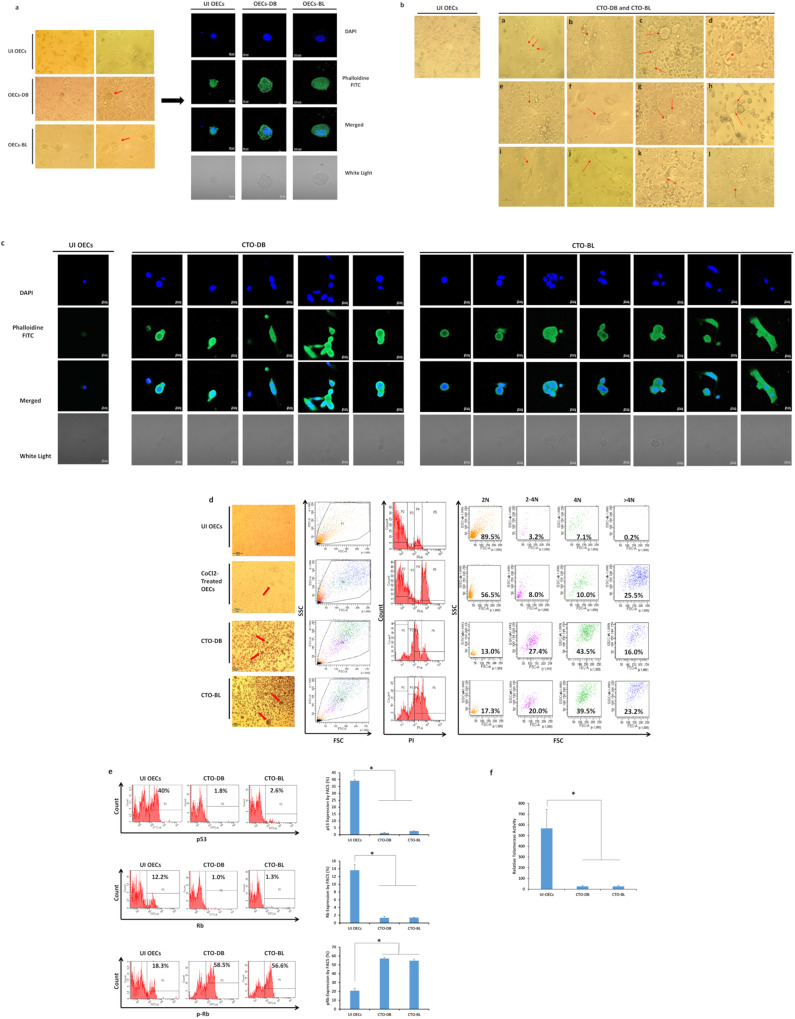
Chronic infection of OECs with the high-risk HCMV clinical isolates and polyploidy detection in OECs cultures. **a** Microscopic images including confocal images of DAPI and phalloidine staining in OECs infected with HCMV-DB and BL; uninfected OECs were used as a negative control. Left panel: magnification ×100 and ×200, scale bar 100 and 50 μm; Right panel: magnification ×63, scale bar 10 μm. Red arrows showing the PGCCs detected in OECs cultures. **b** The appearance of distinct cellular morphologies of the giant cell cycle including (**a**) filopodia, (**b**, **c**, **d**) blastomeres and blastocytes, (**e**) lipid droplets-filled cells, (**f**) multinucleated, (**g**, **h**) budding, (**i**) mesenchymal cells as well as (**j**, **k**, **l**) few atypical morphologies; magnification ×100, scale bar 100 μm. Uninfected OECs were used as a control. **c** Confocal microscopic images of DAPI and phalloidine staining in CTO-DB and BL. Uninfected OECs were used as a negative control; magnification ×63, scale bar 10 μm. **d** Propidium iodide (PI) staining for polyploidy detection in HCMV-transformed OECs. Cobalt chloride (CoCl2)-treated OECs (450 μM) were used as a positive control. Microscopic images of uninfected OECs as well as the PGCCs generated in CTO-DB and BL cultures and post-CoCl2 treatment; magnification ×100, scale bar 100 μm. **e** p53, Rb, and p-Rb expression in uninfected OECs and CTO-DB and BL by FACS. **f** Histograms representing the relative telomerase activity in uninfected OECs as well as CTO-DB and BL. Data are represented as mean ± SD of two independent experiments. *p-value ≤ 0^.^05.

We noticed the existence of large-sized cells having large nuclei that were detected only in OECs chronically infected with the high-risk HCMV-DB and BL strains compared to uninfected OECs (Fig. 2a) and OECs chronically infected with HCMV-KM and FS strains (Supplementary Fig. 1b). Further, around three months post-infection, cellular survival was noted only in the chronically infected OECs-DB and BL compared to OECs-KM and FS (Supplementary Fig. 3). The emerging cells were termed *“CMV-Transformed Ovarian epithelial cells*” or CTO similar to the transformed cells that were previously reported by our group, namely, “CMV-Transformed Human mammary epithelial cells” or CTH cells and “CMV-Elicited GlioBlastoma Cells” or CEGBCs [30, 34].

After applying a morphology-based classification, cellular heterogeneity was detected in CTO-DB and BL cultures including giant cells, blastomeres, blastocytes, multinucleated, mesenchymal, budding, and lipid droplets-rich cells as well as cells exhibiting filopodia and cytoplasmic vacuolization (Fig. 2b). Some of the aforementioned morphologies were further confirmed by confocal microscopy showing mainly multinucleated and mesenchymal cells, asymmetric division, budding, and cells with giant nuclei (Fig. 2c). A high percentage of tetraploidization and polyploidy cells was detected in CTO-DB and BL compared to UI OECs (Fig. 2d). CTO-DB and BL populations were classified into PGCCs (≥4 N), intermediate cells (ICs of 2–4 N), and small cells (SCs of 2 N) (Fig. 2d). As a positive control, cobalt chloride (CoCl_2_) was used to induce PGCCs formation in OECs cultures (Fig. 2d, Supplementary Fig. 4).

Since the blockade of tumor suppressors and decreased telomerase activity have been correlated with tetraploidization in several human cancers [24], p53 and Rb expression as well as telomerase activity were assessed in uninfected OECs, CTO-DB, and CTO-BL (Fig. 2e, f). Downregulation of p53 and Rb proteins was noticed in CTO-DB and BL compared to controls (*p*-value _(UI OECs:CTO-HCMV)_ = 0^.^03), unlike pRb which was upregulated in CTO-DB and BL (*p*-value _(UI OECs:CTO-HCMV)_ = 0^.^03) (Fig. 2e). Notably, telomerase activity was relatively low or undetectable in CTO-DB and BL compared to uninfected OECs (*p*-value = 0^.^03) (Fig. 2f). Hence, p53 and Rb downregulation along with decreased telomerase activity are sufficient to drive polyploidization in CTO-DB and BL, as previously reported [24].

### CTO cells display dedifferentiation, stemness, and EMT characteristics

**Fig. 3:**
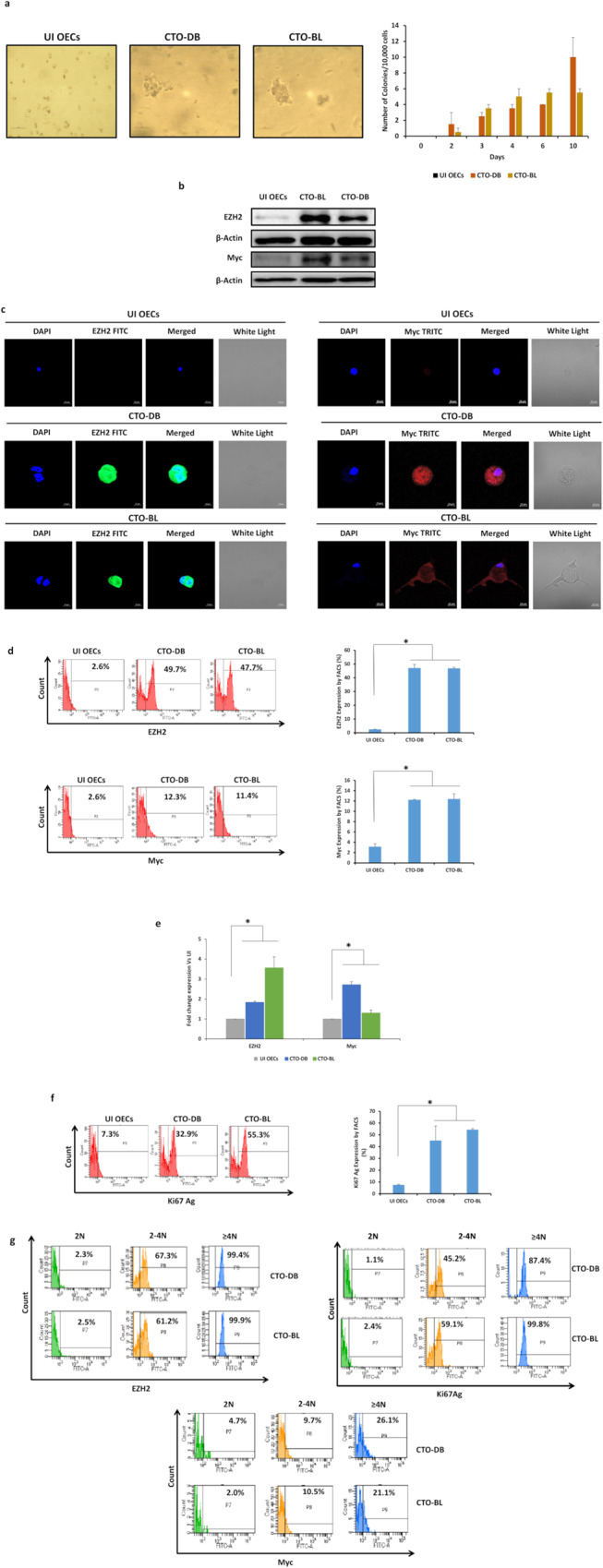
Colony formation in soft agar and the phenotypic characterization of HCMV-transformed OECs. **a** Colony formation in soft agar seeded with CTO-DB and BL (MOI = 1); UI OECs were used as a negative control. Formed colonies were observed under an inverted light microscope (Magnification ×200, scale bar 50 µm). Histogram representing the colony quantification/10,000 cells over days. **b** Immunoblotting data of EZH2 and Myc in uninfected OECs lysates and CTO-DB and BL. β-actin was used as loading control. **c** Confocal microscopic images of EZH2, Myc, and DAPI staining in CTO-DB and BL. UI OECs were used as controls; magnification ×63, scale bar 10 μm. **d** FACS staining of EZH2 and Myc in uninfected OECs as well as CTO-DB and BL. **e** EZH2 and Myc transcripts detection by RT-qPCR. **f** FACS staining of Ki67Ag in uninfected OECs as well as CTO-DB and BL. **g** EZH2, Myc, and Ki67Ag expression in CTO-DB and BL subpopulations (2 N, 2–4 N, and ≥4 N). Data are represented as mean ± SD of two independent experiments. *p-value ≤ 0^.^05.

CTO-DB and BL were seeded in soft agar to assess their oncogenic transforming potential. Colony formation was detected in the cultures seeded with CTO-DB and BL, compared to uninfected OECs (Fig. 3a). On the proteomic level, EZH2 and Myc upregulation was detected in CTO-DB and BL compared to uninfected OECs (*p*-value _(UI OECs:CTO-HCMV)_ = 0^.^03) (Fig. 3b–d); on the contrary, a limited EZH2 and Myc upregulation was observed in OECs-KM and FS (*p*-value _(UI OECs:OECs-HCMV)_ = 0^.^02) (Supplementary Fig. 1c). A significant positive correlation between EZH2 and Myc protein expression was detected in CTO cells (*r* = 0.983, *p*-value < 0^.^001) (Supplementary Fig. 5). Further, EZH2 and Myc transcripts were upregulated in CTO-DB and BL compared to uninfected controls (*p*-value _(UI OECs:CTO-HCMV)_ = 0^.^03) (Fig. 3e). Altogether, a remarkable EZH2 upregulation parallel to the limited increase in Myc expression was noticed in CTO infected with the high-risk HCMV-DB and BL strains. EZH2 and Myc were expressed mainly in the PGCCs subpopulation of CTO-DB and BL (Fig. 3g). A limited increase in SUZ12 expression was observed in CTO-DB and BL compared to uninfected OECs (*p*-value _(UI OECs:CTO-HCMV)_ = 0^.^03) (Supplementary Fig. 6a, b). Upon assessing the proliferative potential of OECs chronically infected with HCMV high-risk strains, Ki67Ag was highly prominent in CTO-DB and BL compared to uninfected OECs (*p*-value _(UI OECs:CTO-HCMV)_ = 0^.^03) (Fig. 3f). High Ki67Ag expression was detected in the large cells (Fig. 3g).

**Fig. 4:**
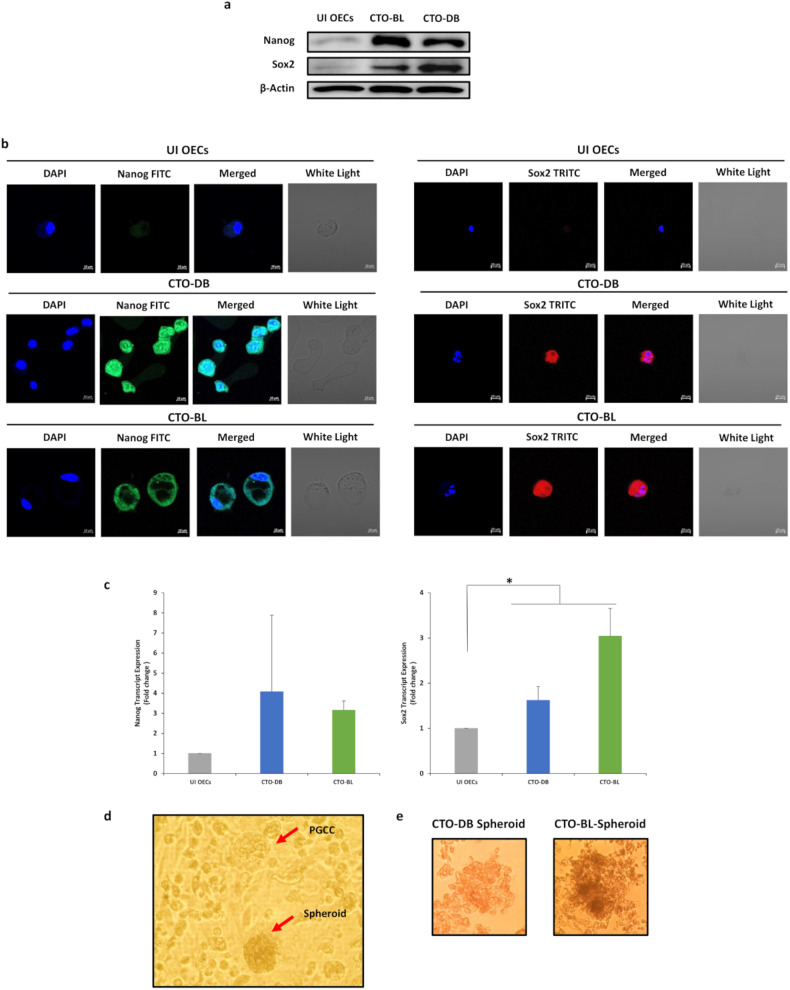
HCMV-transformed OECs display an embryonic stemness phenotype and possess spheroid-forming potential. **a** Immunoblotting data of Nanog and Sox2 in uninfected OECs lysates and CTO-DB and BL. β-actin was used as loading control. **b** Confocal microscopic images of Nanog, Sox2, and DAPI staining in CTO-DB and BL. UI OECs were used as controls; magnification ×63, scale bar 10 μm. **c** Nanog and Sox2 transcripts detection by RT-qPCR. Data are represented as mean ± SD of two independent experiments. **d** Spontaneous spheroid formation and PGCCs were detected under an inverted light microscope in HCMV-transformed OECs cultures. Magnification ×100, scale bar 100 μm. **e** Spheroid generation from the chronically infected DB and BL OECs in methyl-cellulose assay; magnification ×100, scale bar 100 µm. *p-value ≤ 0^.^05.

CTO-DB and BL displayed an embryonic stemness phenotype. The key regulatory genes maintaining the pluripotency and self-renewal properties of embryonic stem cells, Nanog, Sox2, and Oct4 were shown to be highly expressed in CTO-DB and BL compared to uninfected OECs (Fig. 4a, b, and Supplementary Fig. 7a). Additionally, transcripts of Nanog, Sox2, and Oct4 were elevated in CTO-DB and BL (Nanog, *p*-value _(UI OECs:CTO-HCMV)_ = 0^.^06; Sox2 and Oct4, *p*-value _(UI OECs:CTO-HCMV)_ = 0^.^03) (Fig. 4c and Supplementary Fig. 7b). Besides PGCCs appearance in CTO-DB and BL cultures, it’s worth mentioning the appearance of spontaneous spheroids in CTO-BL cultures (Fig. 4d). Further, in the presence of methylcellulose, spheroids were generated in CTO-DB and BL cultures (Fig. 4e). Finally, CD44, a marker of stemness and invasiveness, was upregulated in CTO-DB and BL cultures compared to uninfected OECs (Supplementary Fig. 8).

**Fig. 5:**
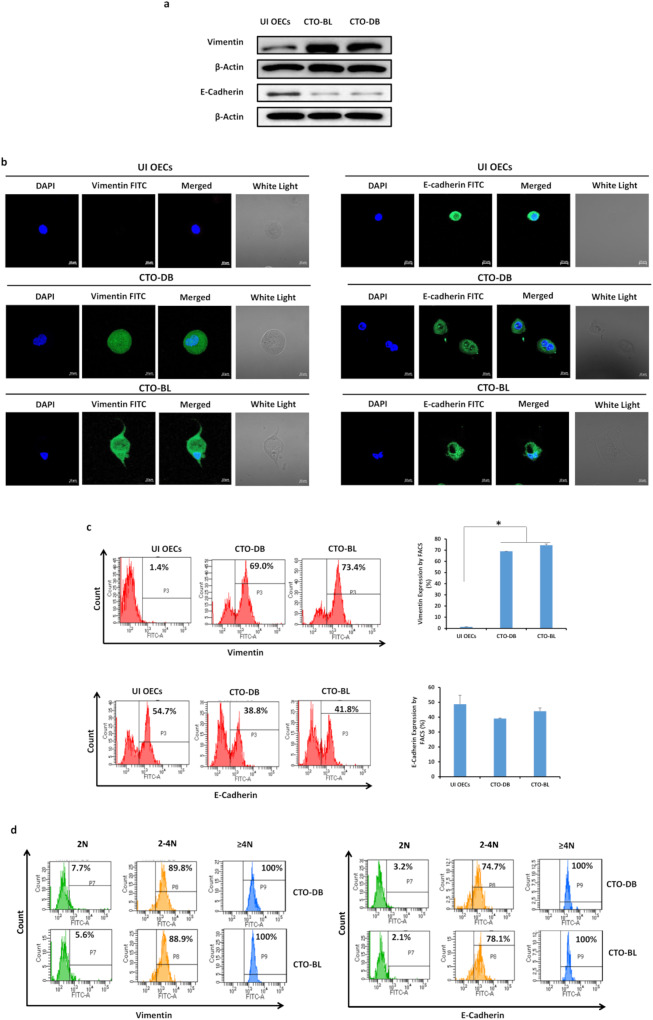
HCMV infection of OECs enhances EMT/MET hybrid traits. Vimentin and E-cadherin expression by western blot (**a**), confocal microscopy (**b**), and FACS (**c**) in CTO-DB and BL. Uninfected OECs were used as controls. **d** Vimentin and E-cadherin expression in CTO-DB and BL subpopulations (2 N, 2–4 N, and ≥4 N). Data are represented as mean ± SD of two independent experiments. *p-value ≤ 0^.^05.

EMT fuels cancer progression, tumor cell invasion, and therapy resistance [35]. PGCCs gain strong invasiveness and migration ability after they undergo EMT [36]. Vimentin was strongly upregulated in DB and BL-infected OECs (*p*-value _(UI OECs:CTO-HCMV)_ = 0^.^03), whereas a slight decrease in E-cadherin was noticed compared to uninfected OECs (*p*-value _(UI OECs:CTO-HCMV)_ = 0^.^06) (Fig. 5a–c). Vimentin and E-cadherin were expressed mainly in the PGCCs and ICs subpopulations of CTO-DB and BL (Fig. 5d). Altogether, the co-existence of vimentin and E-cadherin was detected in CTO-DB and BL, indicating a mesenchymal/epithelial hybrid state that was also accompanied with occasionally existing small cells possessing an elevated E-cadherin expression (Fig. 5 and Supplementary Fig. 9). The co-existence of mesenchymal and epithelial phenotypes confirms the cellular plasticity of CTO-DB and BL. As a control, EZH2, Myc, Ki67Ag, Vimentin, E-cadherin, and CD44 protein expression in the subpopulations of uninfected OECs was provided in Supplementary Fig. 10.

### Lytic and latent HCMV replication in transformed OECs

**Fig. 6:**
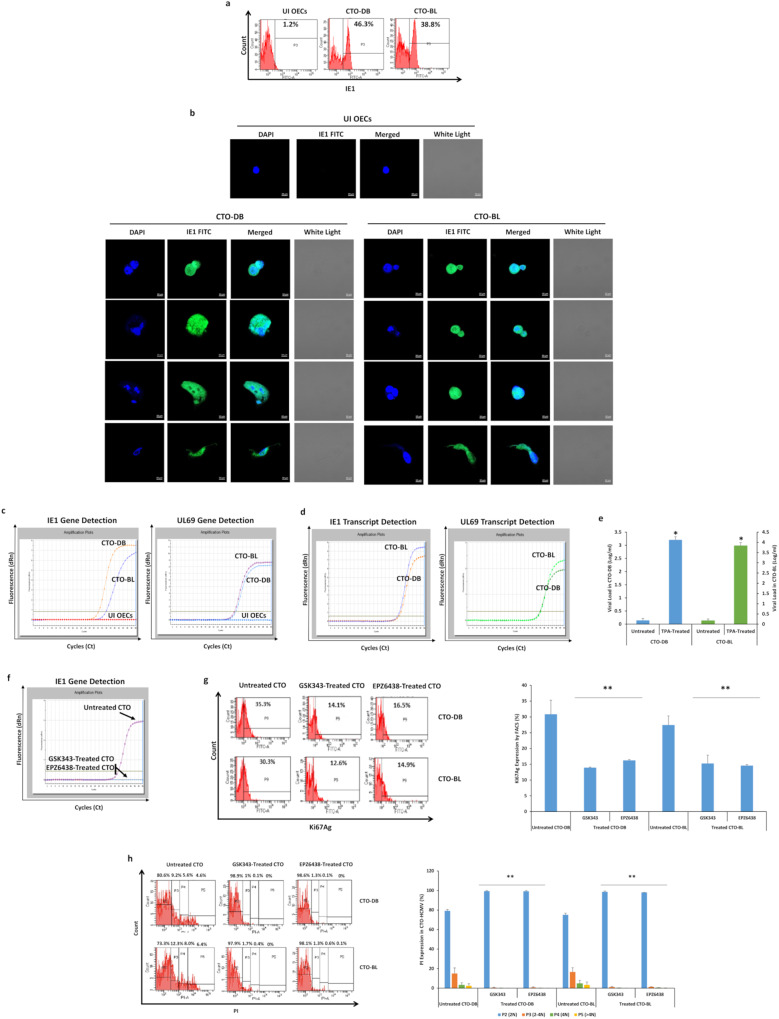
Sustained HCMV replication in chronically HCMV-infected OECs. **a** IE1 expression by FACS in chronically infected OECs-DB and BL cultures. **b** IE1 expression by confocal microscopy in CTO-DB and BL; uninfected OECs were used as a control. Nuclei were counterstained with DAPI; magnification ×63, scale bar 10 μm. **c** IE1 and UL69 gene detection in chronically infected OECs-DB and BL as measured by qPCR. Uninfected OECs were used as a negative control. **d** IE1 and UL69 transcripts detection as measured by RT-qPCR. **e** Histogram representing the viral load post-TPA treatment in CTO-DB and BL cultures as measured by IE1-qPCR. **f** IE1 gene detection by qPCR in untreated CTO-DB, and CTO-DB treated with two EZH2 inhibitors (0.1 µM of GSK34 and EPZ6438). Ki67Ag expression (**g**) and PI staining (**h**) in untreated CTO-DB/BL and CTO-DB/BL treated with 0.1 µM of GSK34 and EPZ6438 by FACS. Data are represented as mean ± SD of two independent experiments. *p-value ≤ 0^.^05; **p-value ≤ 0^.^01.

Sustained HCMV replication was confirmed in OECs chronically infected with HCMV-DB and BL, namely CTO-DB and BL (Fig. 6). IE1 protein was remarkably detected in CTO-DB and BL versus uninfected OECs (Fig. 6a, b); elevated IE1 expression was detected mainly in the PGCCs subpopulation compared to ICs and SCs (Supplementary Fig. 11). IE1 and UL69 genes and transcripts were detected in CTO-DB and BL compared to uninfected OECs (Fig. 6c, d), indicating lytic HCMV replication. In addition, HCMV latency in CTO-DB and BL cultures was established by IE1 reactivation that was observed post-TPA treatment (*p*-value _(CTO:TPA treated CTO)_ = 0^.^02) (Fig. 6e and Supplementary Fig. 12).

Given that EZH2 is considered a major tumor marker and an effective therapeutic target for OC, herein, we evaluated the impact of two EZH2 inhibitors (GSK343 and EPZ6438) on CTO proliferation and polyploidization. Upon EZH2 blockade (Supplementary Fig. 13), detection of HCMV-IE1 gene was suppressed (Fig. 6f). Ki67Ag protein expression was decreased post-EZH2 inhibitor treatment of CTO-DB and BL compared to untreated CTO cells (*p*-value _(CTO:EZH2 inhibitors treated CTO)_ = 0^.^004) (Fig. 6g). Upon assessing the PI expression in the subpopulations of CTO cells, GSK343 and EPZ6438 reduced tetraploidization and polyploidization in treated CTO cells compared to controls (*p*-value _(CTO:EZH2 inhibitors treated CTO)_ = 0^.^007 and 0^.^004, respectively) (Fig. 6h).

### EZH2 upregulation in HCMV-positive HGSOC biopsies and the isolation of three oncogenic HCMV strains from EZH2^High^ HGSOC biopsies

**Fig. 7:**
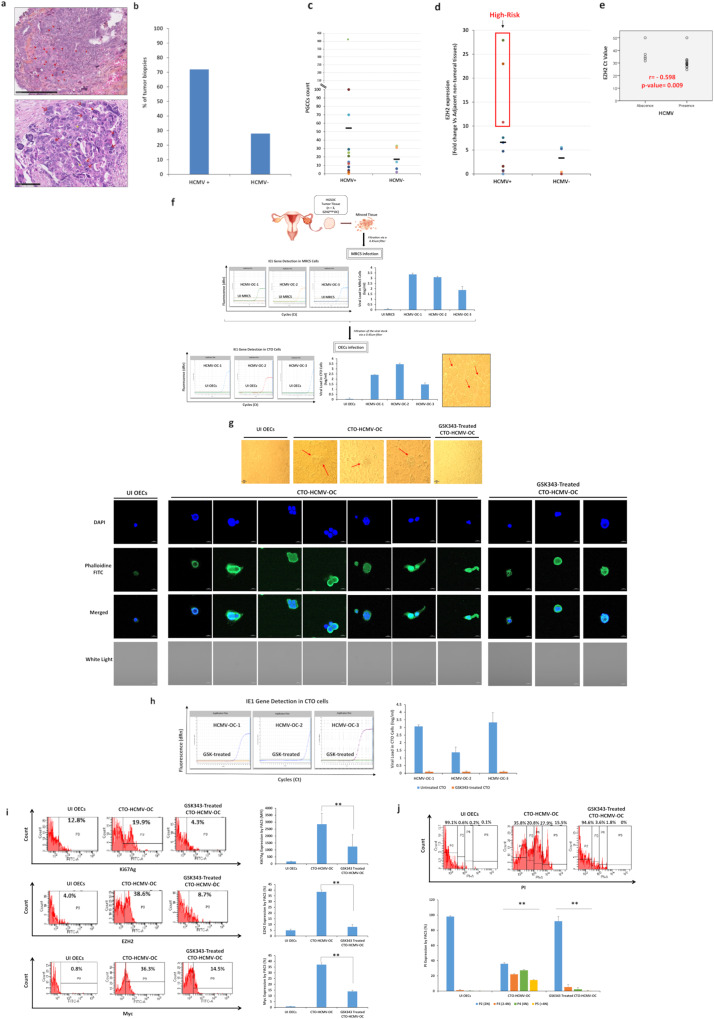
HCMV detection, PGCCs presence as well as EZH2 expression in ovarian cancer biopsies. **a** Ovarian cancer tissue HES staining; magnification ×400, scale bar 250 µm (upper panel) and 100 µm (lower panel). Red arrow representing the PGCCs while the yellow arrow represents the diploid carcinoma cells. **b** Histogram representing HCMV presence in the ovarian tumor biopsies. **c** Scattered plots showing the PGCCs count in HCMV-positive and negative ovarian tumor biopsies. **d** Scattered plots representing EZH2 expression in HCMV-positive and negative ovarian tumor biopsies by RT-qPCR. Red box indicates the high-risk HCMV strains with high EZH2 expression. **e** Correlation test between Ct value of EZH2 and HCMV presence *p*-values were determined by Spearman’s correlation test. **f** Isolation protocol of the three high-risk HCMV-ovarian cancer strains from HGSOC tissues; histogram representing the viral replication of the isolated HCMV strains in MRC5 cells and CTO cultures by IE-qPCR. CTO cells were observed under an inverted light microscope (magnification ×200, scale bar 50 µm). **g** Light microscopic images (magnification ×200, scale bar 50 µm) as well as confocal images of DAPI and phalloidine staining in CTO-HCMV-OC and GSK343-treated CTO cells (magnification ×63, scale bar 10 μm); uninfected OECs were used as a negative control. **h** IE1 gene detection in CTO-HCMV-OC and GSK343-treated CTO cells by IE-qPCR. **i**, **j** Ki67Ag, EZH2, Myc expression (**i**) and PI staining (**j**) in CTO-HCMV-OC, GSK343-treated CTO cells, and uninfected OECs by FACS. Data are represented as mean ± SD of two independent experiments. **p-value ≤ 0^.^01.

To further assess the role of HCMV, EZH2, and PGCCs induction in vivo, we analyzed 25 OC biopsies (HGSOC biopsies *n* = 18 and adjacent non-tumoral biopsies *n* = 7) (Supplementary Table 3) for the presence of HCMV, PGCCs count, as well as EZH2, Myc, and Akt expression (Fig. 7, Supplementary Fig. 14, and Supplementary Fig. 15). PGCCs with giant or multiple nuclei were detected in the HGSOC biopsies (Fig. 7a). HCMV was detected in 72% of HGSOC biopsies (Fig. 7b). Elevated PGCCs count was mainly detected in HCMV-positive HGSOC biopsies (Fig. 7c). HCMV-positive tumor biopsies displayed mostly an enhanced EZH2 and Akt with a limited Myc expression (Fig. 7d, Supplementary Fig. 14, and Supplementary Fig. 15a). A significant positive correlation was found between HCMV presence and EZH2 as well as Akt expression (*r* = −0.598, *p*-value = 0^.^009 and *r* = − 0.466, *p*-value = 0^.^05, respectively, based on Ct values) (Fig. 7e and Supplementary Fig. 15b). A significant positive correlation was detected between Akt expression and EZH2 as well as Myc expression (*r* = 0.420, *p*-value = 0^.^015 and *r* = 0.620, *p*-value = 0^.^006, respectively) (Supplementary Fig. 15b). Among the eighteen HCMV-positive HGSOC biopsies, three biopsies with the highest EZH2 expression were considered for HCMV isolation. The three HCMV-OC strains were isolated by tissue disruption and filtration, and were subsequently grown in MRC5 cells showing a peak of viral load of 3 logs around day 7 post-infection (Fig. 7f). Following OECs infection with the three HCMV-OC strains, HCMV presence was confirmed by the detection of IE1 gene in addition to the morphological changes that appeared in the OECs infected cultures, for instance, giant and multinucleated cells showing cell budding as well as mesenchymal cells (Fig. 7f, g). Post-EZH2 inhibition, using GSK343, no HCMV replication was detected in addition to the absence of morphological heterogeneity in the infected cultures (Fig. 7g, h). Upregulation of Ki67Ag, EZH2, and Myc expression was observed in CTO-HCMV-OC compared to uninfected OECs and GSK-treated CTO-HCMV-OC cells (Ki67Ag *p*-value _(CTO:GSK343-CTO)_ = 0^.^01; EZH2 *p*-value _(CTO:GSK343-CTO)_ = 0^.^002; Myc *p*-value _(CTO:GSK343-CTO)_ = 0^.^002) (Fig. 7i). GSK343 reduced Ki67Ag, EZH2, and Myc expression by 56%, 79%, and 63%, respectively (Fig. 7i). EZH2 inhibition reduced PGCCs percentage in infected cultures compared to untreated CTO cells (*p*-value = 0^.^002) (Fig. 7j).

## Discussion

**Fig. 8:**
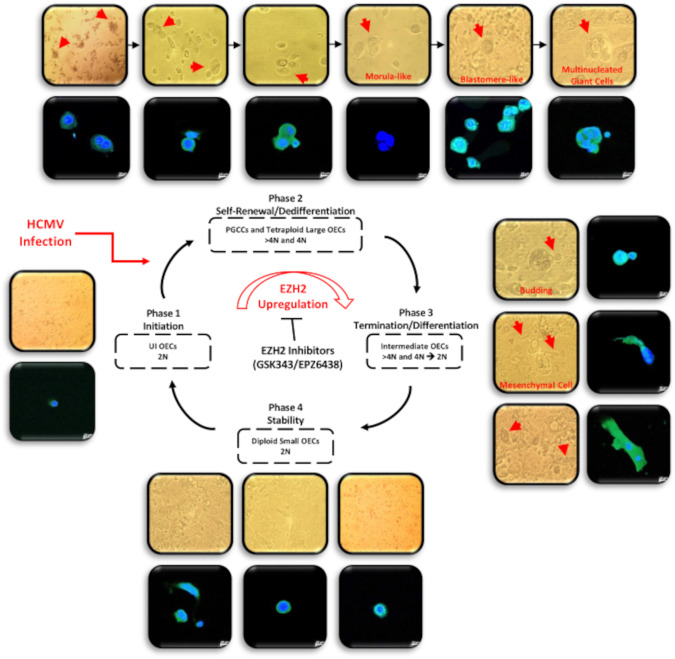
A schematic representing the giant cell cycling following HCMV infection of OECs. Giant cell cycle representing four distinct phases including initiation, self-renewal, termination and stability. Upon HCMV infection, the 2 N OECs go into the initiation phase through endoreplication. Polyploid cells ( > 4 N) and tetraploid cells (4 N) generate in the self-renewal/dedifferentiation stage due to HCMV infection and the subsequent EZH2 upregulation. Cell budding takes place from multinucleated or mononucleated giant cells generating intermediate 2–4 N OECs during the termination/differentiation phase. Intermediate OECs gradually reach stability and are converted into diploid small OECs (2 N).

In the present study, we assessed the potential transforming capacities of the high-risk HCMV-DB and BL strains, following the OECs infection. The OECs infection with the high-risk HCMV-DB and BL strains resulted in a pro-oncogenic cellular environment and sustained growth of CMV-transformed OECs with soft agar colonies formation. The CTO cells dedifferentiated, displayed stemness as well as EMT-MET hybrid phenotype, and finally resulted in PGGCs generation (Fig. 8) and spheroid formation. HCMV presence accompanied by polyploidy, EZH2 upregulation, and malignant phenotype potentially confirm the transformation process. In vivo, 72% of HGSOC biopsies were found to harbor HCMV with elevated PGCCs count as well as enhanced EZH2 expression, revealing a strong correlation between HCMV, PGCCs, and EZH2 expression. Three HCMV-OC strains were isolated from EZH2^high^ OC tumors that transformed OECs toward CTO possessing increased EZH2, Ki67Ag, and Myc expression parallel to polyploidy induction. The expression of the aforementioned markers and polyploidy were curtailed by EZH2 inhibitors therapy.

PGCCs play a fundamental role in tumor progression and in regulating tumor heterogeneity [37, 38]. Accumulating evidence reveals the presence of PGCCs in OC, particularly the HGSOC, where PGCCs act as stem-like, self-renewing cells that are considered prognostic factors for OC [38, 39]. In our study, CTO cells generated PGCCs, and were heterogeneous showing distinct morphological features including budding, filopodia, lipid droplets-filled cells, blastomere-like cells, and multinucleated cells that were previously detected in OC especially HGSOC [13, 37, 40–44] and reported upon HCMV infection of human mammary epithelial cells [17–19]. Our findings indicated that polyploidy harboring HCMV might induce the acquisition of a malignant phenotype via the giant cell cycling [14]. p53 and Rb inactivation have been correlated with tetraploidization in human tumors [22]. Indeed, p53 was shown to be mutated in more than 50% of human tumors, especially HGSOC; most of the p53 mutations acquired an oncogenic function. It’s worth mentioning that tumor-promoting viruses reduce p53 activity [45]. Studies have shown that the incidence of tetraploidy occurs in p53-deficient cells possessing prolonged DNA damage due to persistent telomere dysfunction [46]. p53 and Rb expression was decreased in CTO-DB and BL that showed high percentages of tetraploidization and decreased telomerase activity, in line with the telomere-driven tetraploidization induced by critically short telomeres with the potential to promote tumorigenesis in early cancerous lesions [24]. Several HCMV products have been involved in the cellular transformation including IE1, IE2, pp65, US28, cmvIL-10, UL76, UL44, and UL84, etc. Such expression of HCMV gene products could impair the pathways of p53 and Rb [6, 8]. HCMV-IE1, IE2, and UL97 allow the evasion of p53 and Rb [8]. Hence, the impaired p53 and Rb pathways in CTO cells were due to the transforming potential of HCMV contrasting the Ras, human telomerase reverse transcriptase (hTERT), and SV40-mediated transformation of human ovarian cells [47].

Numerous biomarkers have become essential in characterizing and managing ovarian cancers [48]. EZH2, a downstream target of Myc oncogene, has been involved in regulating cell cycle progression [49]. Elevated EZH2 activity has been reported in approximately 85% of epithelial ovarian carcinomas [50]. Since EZH2 enhances cell proliferation, inhibits apoptosis, promotes angiogenesis, metastasis and therapy resistance in OC [50, 51], inhibiting EZH2 suggests an effectual strategy for developing OC therapies. A study reported that EZH2 degradation profoundly blocked ovarian tumor cell proliferation and tumorigenesis in vitro and in vivo [52]. Given the recent approval of the EZH2-targeting agent (tazemetostat) by the FDA as a treatment for follicular lymphoma, enhanced therapies may soon be available for suppressing ovarian cancers [50]. A combination of EZH2 inhibitor, PARP inhibitor, and/or immune checkpoint blockers might be synergistic in ovarian cancers [53, 54]. In line with the critical role of EZH2 in epithelial ovarian cancer [25], EZH2 overexpression was detected in CTO-DB and BL compared to uninfected OECs. EZH2 inhibition restricted the proliferation potential of CTO cells and limited tetraploidization. Moreover, Myc, as a transcription factor, plays an essential role in regulating multiple cellular processes. Enhanced Myc expression and/or mutated Myc have been reported in most human cancers and have been observed in 20–50% of ovarian carcinoma [55, 56]. A limited expression of Myc was detected in the PGCCs population of HCMV-infected OECs, reinforcing the emerging data on limited Myc expression in dormant cells [57]. EZH2 upregulation was shown to be the main feature in CTO cells and ovarian epithelial cancers. Anti-EZH2 treatment makes EZH2 a desirable therapeutic target, unlike anti-Myc therapeutics. Given the absence of enzymatic activity and the lack of surface domains specific for most pharmacological inhibitors, Myc is considered an “undruggable” protein [55], hence anti-Myc therapeutics might fall short of expectations in limiting ovarian cancers.

Cells possessing sphere-forming potentials were shown to reside in the malignant ascites of OC patients [58–60]. In OC, the aforementioned cells are strong contributors to tumor progression, metastasis, chemotherapy resistance, and disease relapse [59]. The spontaneous spheroid generation along with the high expression of Nanog and Oct4 in CTO-DB and BL is in line with the previously described OC cell phenotype [61]. Given their role in maintaining pluripotency and long-term self-renewal, the embryonic transcription factors Oct4 and Nanog are recognized as part of the stem cell signature that strongly correlates with spheroid formation, tumor initiation, and chemoresistance in ovarian cancer cells [61, 62]. CD44 was highly expressed in CTO-DB and BL cells; it has been previously shown to drive the progression of several tumors [63, 64], maintain stem cell quiescence, and promote EMT in OC [65]. Further, CD44 has been linked to the sphere-forming, self-renewing and tumor-initiating potential of OC cells [66]. The co-existence of vimentin and E-cadherin in CTO-DB and BL highlights the high cellular plasticity. EMT plasticity with EMT and MET alternately taking place was revealed during HGSOC progression where cells co-express epithelial and mesenchymal determinants [44, 67, 68]. Besides cellular plasticity, the hybrid E/M state promotes stem-like properties as well as metastatic and tumorigenic potential [69, 70].

Upon infecting OECs with HCMV-DB and BL strains, IE1 and pp65 proteins were detected which is in line with the recent studies that have identified high expressions of HCMV-IE or pp65 in ovarian tumor samples that were associated with poor survival outcomes [1, 33], suggesting that HCMV infection could potentially promote cancer progression. All in all, HCMV-induced EZH2 expression along with the embryonic stem-like phenotype and cellular plasticity in the IE1-expressing OECs/CTO cells could establish a significant model in the context of OC. Several studies shed light on the existence of herpesviruses DNA and proteins in ovarian tissues that may hold potential in the process of OC tumorigenesis [1, 33, 71]. Herein, we reported the detection of HCMV in HGSOC biopsies displaying elevated PGCCs count as well as enhanced EZH2 expression. It is worth mentioning that, neither HPV nor EBV was detected in the eighteen tested HGOSC biopsies.

Three HCMV strains with a strong transforming potential, so called high-risk strains, were isolated from EZH2^High^ HGOSC biopsies. After OECs infection, CTO cells were generated with morphological features matching the previously described CTO-DB and BL. Additionally, we evaluated EZH2 expression in the high-risk HCMV strains that were recovered directly from HGSOC biopsies thereby assessing their oncogenic and stemness potential. Unlike GSK-treated CTO, the expression of EZH2, Myc, and Ki67Ag was predominantly elevated in untreated CTO-HCMV-OC cells parallel to the PGCCs appearance. In summary, HCMV clinical strains reside in HGOSC biopsies retaining cancer-promoting potentials.

The high-risk HCMV strains that were isolated from HGSOC, TNBC, and GBM patients shared the same transforming potential [17, 30, 34]. PGCCs were detected in the aforementioned cell cultures that were shown to highly express Myc and EZH2 pointing toward a potential link between HCMV, PGCCs, EZH2, and Myc. CTO and CTH cells dedifferentiated and revealed stemness as well as EMT traits [17, 19, 34]. On the other hand, CEGBCs dedifferentiated and displayed stemness, PMT as well as invasiveness features [30]. The phenotypic changes were similar in HCMV-infected ovarian and mammary epithelial cells. However, the difference was mainly the detection of high percentages of tetraploid cells in the CTO cultures compared to the >4 N population detected in CTH cells. In both models, a high degree of cellular plasticity was detected knowing that ovarian and mammary epithelial cells are isolated from human glandular organs.

Given that cancer patients with lower HCMV activity have a better prognosis, targeting HCMV might have a vital role in treating OC [33]. Post-anti-CMV therapy, a remarkably high extended survival was observed in both newly diagnosed and recurrent glioblastoma patients [72]. Current vaccine candidates have focused on several HCMV antigens/epitopes such as gB, gH, pentamer complex, pp65 and IE1 [73, 74]. Recently, CMV-specific immunotherapy including cytotoxic T lymphocyte (CTL) or dendritic cell (DC)-based vaccines has contributed to minimal successful outcomes in glioblastoma treatment [74, 75]. In addition, using EZH2 inhibitors to specifically target ovarian tumors could ultimately improve patients outcomes [25]. Two inhibitors triggering EZH2 degradation (DZNep and YM281) exhibited potent efficacy in OC cell lines and xenografts [52]. GSK343 significantly induced apoptosis and inhibited the invasion of ovarian epithelial cells in 3D cultures which more closely mimics the tumor microenvironment in vivo [76]. In patient-derived glioma stem cells, GSK343 was shown to suppress the stemness traits [77]. Interestingly, in the present study, EZH2 inhibitors totally blocked HCMV replication (Figs. 6f and 7h). It’s worth noting that the HCMV major immediate early promoter (MIEP) transcriptional repressor, growth factor independence1 (GFI1), is controlled by the EZH2-NDY1/KDM2B-JARID2 axis. Therefore, EZH2 inhibitors might result in an enhanced GFI1 expression which could block viral replication [78]. Hence, combinational therapies including EZH2 inhibitors may prove to be a promising milestone in developing therapeutic strategies for ovarian cancer treatment.

## Conclusion

In conclusion, our data indicated that the high-risk HCMV strains which triggered EZH2 upregulation can induce polyploidy, OECs dedifferentiation, stemness, and EMT/MET characteristics parallel to the existence of giant cell cycling. Future studies, with more detailed analyses, may establish new avenues to understand the complex pathogenesis of OC and might pave the way for novel targeted therapies.

## Material and methods

### Cell cultures

Human ovarian surface epithelial cells (HOSE cells or OECs, as mentioned in our study) and human embryonic lung fibroblasts (MRC5 cells) were purchased from Innoprot (Derio, Spain) and RD-Biotech (Besançon, France), respectively. OECs were cultivated in ovarian epithelial cell medium (serum-free) supplemented with ovarian epithelial cell growth supplement (OEpiCGS) and penicillin/streptomycin solution (P60132, Innoprot). MRC5 cells were cultivated in Dulbecco’s modified Eagle medium (Sigma-Aldrich, Saint-Louis, MO) supplemented with 10% fetal bovine serum (Dutscher, Bernolsheim, France) and penicillin (100 U/mL)-streptomycin (100 μg/mL) (Life Technologies, Eugene, OR). Cells were cultured under standard conditions (37 °C, 5% CO_2_, 95% humidity). To note that, HOSE cells or OECs were isolated from healthy human ovaries, as mentioned in the technical data sheet (P10982, Innoprot). Cultures were verified as mycoplasma-free as determined by monthly screenings (VenorGem classic mycoplasma detection, Minerva Biolabs).

### Characterization of HCMV clinical isolates

Clinical HCMV strains, namely HCMV-DB (GenBank KT959235), BL (GenBank MW980585), KM, and FS were isolated from patients that were hospitalized at Besançon University Hospital (France) as previously described [19, 34]. Cell-free virus stocks and infections were performed as previously detailed. It is worth mentioning that careful screening of our viral stocks was conducted to rule out the presence of other oncoviruses [19].

### Viral growth and detection

Infections of OECs, quantification of viral replication, and HCMV detection were performed as previously described [19]. OECs (1 × 10^6^) infection with the clinical isolates was performed at a multiplicity of infection (MOI) of 1. For HCMV quantification, cell-free infectious supernatant was collected, DNA was isolated (EZNA Blood DNA Kit, D3392-02, Omega BIO-TEK, Norcross, GA) and real-time IE1 quantitative PCR (qPCR) was performed using KAPA SYBR FAST Master Mix (KAPA BIOSYSTEMS, Potters Bar, UK) and IE1 and UL69 primers. Real-time qPCR reactions were activated at 95 °C for 10 min and then 50 cycles (15 s at 95 °C and 1 min at 60 °C) were conducted using a Stratagene Mx3005P thermocycler (Agilent Technologies, Santa Clara, CA). Results collection and analysis were done using MxPro qPCR software. Primers used are listed in Supplementary Table 1.

### Western blotting

IE1, pp65, Myc, EZH2, Sox2, Nanog, E-cadherin, and vimentin expression in uninfected OECs, HCMV-infected OECs and CTO cells was assessed as described previously [34]. β-actin was used as a loading control. Antibodies used are supplied in Supplementary Table 2.

### Flow cytometry analysis

Cells (1 × 10^5^) were collected from uninfected OECs, HCMV-infected OECs and CTO cells. Cells were fixed, permeabilized, and stained as previously reported [19]. Cytofluorometric analysis was achieved using a BD LSRFortessa X-20 (BD Biosciences) flow cytometer. FACSDiva software (BD Biosciences) was used for data collection and analysis. The antibodies used are provided in Supplementary Table 2. For cell cycle analysis, uninfected OECs or CTO cells were washed in 1X PBS, fixed in 70% ethanol, and resuspended in 50 µg/ml propidium iodide (P3566, life technologies, Eugene, USA) with 0.1 mg/ml RNase (R4642, Sigma-Aldrich, Saint-Louis, MO, USA), then incubated at 37 °C for 30 min as described previously [17].

### Assessment of telomerase activity

Uninfected OECs and CTO cells were collected, washed with PBS, and resuspended in RIPA lysis buffer and 4% protease inhibitor on ice for 30 min. Samples were then centrifuged at 16,000 g for 30 min at 4 °C and the protein concentration was determined using Bradford assay (DC Protein Assay kit, Bio-Rad Laboratories, Hercules, CA). Telomerase activity was assessed as described previously and according to the manufacturer’s instructions [19].

### Reverse transcription quantitative polymerase chain reaction (RT-qPCR)

The detection of IE1, UL69, EZH2, Myc, Sox2, Nanog, and Oct4 transcripts was assessed by RT-qPCR as detailed previously [79]. Briefly, total RNA was extracted using E.Z.N.A. Total RNA Kit I (Omega Bio-Tech, GA, USA), and reverse transcription was performed using the SuperScript IV First-Strand Synthesis kit (Invitrogen, Carlsbad, CA, USA). The expression of markers was measured by real-time qPCR using a KAPA SYBR FAST Master Mix (KAPA BIOSYSTEMS, KK4601) and specific primers according to the manufacturer’s protocol. Primers used are listed in Supplementary Table 1.

### Confocal microscopy

Confocal microscopy of uninfected and HCMV-infected OECs as well as spheroids was performed as previously detailed [19]. The antibodies used are provided in Supplementary Table 2.

### Soft agar colony formation assay

Colony formation in soft agar (Colorimetric assay, CB135; Cell Biolabs Inc., San Diego, CA) seeded with uninfected and HCMV-infected OECs was performed as previously described [19].

### Spheroid formation assay

Spheroids of OECs were prepared as described previously [80]. Single cells (1 × 10^4^) isolated by accutase were seeded in a serum-free OECs medium containing methylcellulose.

### EZH2 inhibition assay

CTO-DB and BL were treated with 0.1 μM of two EZH2 inhibitors (GSK343 and EPZ6438). Treatment was renewed every 2 days. Ki67Ag and EZH2 expression by FACS was assessed in treated and untreated CTO cells. HCMV-IE1 gene detection was detected in the supernatants of treated and untreated CTO cells.

### Ovarian cancer biopsies

Ovarian biopsies (*n* = 25; HGSOC biopsies *n* = 18, and adjacent non-tumoral biopsies *n* = 7) were provided by the Regional tumor bank (BB0033-00024 Tumorothèque Régionale de Franche-Comté). Clinical data and treatments of the OC patients were provided in Supplementary Table 3. A written informed consent for participation was obtained from all patients. The study was authorized by the local ethics committees of Besançon University Hospital (Besançon, France) and the French Research Ministry (AC-2015-2496, CNIL n°1173545, NF-S-138 96900 n°F2015). Genomic DNA was isolated from patient biopsies, and HCMV presence was identified by qPCR using specific primers against IE1 and UL69 genes. RNA was extracted from the biopsies and reverse transcription was performed as reported previously [17, 79]. Expression of EZH2, Myc, Akt, and β-2-Microglobulin was assessed by real-time qPCR using specific primers according to the manufacturer’s protocol. Fold change expression in OC biopsies versus adjacent non-tumoral biopsies was calculated using the delta-delta Ct method [17, 79]. Neither human papillomavirus (HPV) nor Epstein-Barr virus (EBV) was detected in the ovarian cancer biopsies. Primers used are listed in Supplementary Table 1. PGCCs presence in HGSOC biopsies was confirmed by hematoxylin and eosin staining; briefly, PGCCs were counted in five hot spots of each tumor sample (magnification ×400, field diameter of 250 µm and 100 µm, Zeiss Axiostar). Three HCMV-OC strains were isolated from HGSOC biopsies.

### Statistical analysis

Quantitative results are reported as mean ± SD of independent experiments. Statistical analyses were done using Wilcoxon and Mann*-*Whitney tests; a *p*-value ≤ 0^.^05 was considered to be statistically significant [*: ≤0^.^05; **: ≤0^.^01; ***: ≤0^.^001]. Correlation analysis was done using Spearman, Pearson, and Kendall’s Tau correlation tests. Microsoft Excel was used to construct the plots and histogram data.

### Supplementary information

Supplementary data

## Data Availability

The data supporting the findings of this study are available within the article and its Supplementary Information files and from the corresponding authors on request.
