# Supplementary material for: Polyploidy, EZH2 upregulation, and transformation in cytomegalovirus-infected human ovarian epithelial cells
Source: Oncogene. 2023 Aug 26;42(41):3047–61. doi: 10.1038/s41388-023-02813-4 (PMC10555822; doi:10.1038/s41388-023-02813-4)
Supplement: Supplementary file 1 — Supplementary data [file 41388_2023_2813_MOESM1_ESM.docx]

**Supplementary Data**

# Supplementary Figures


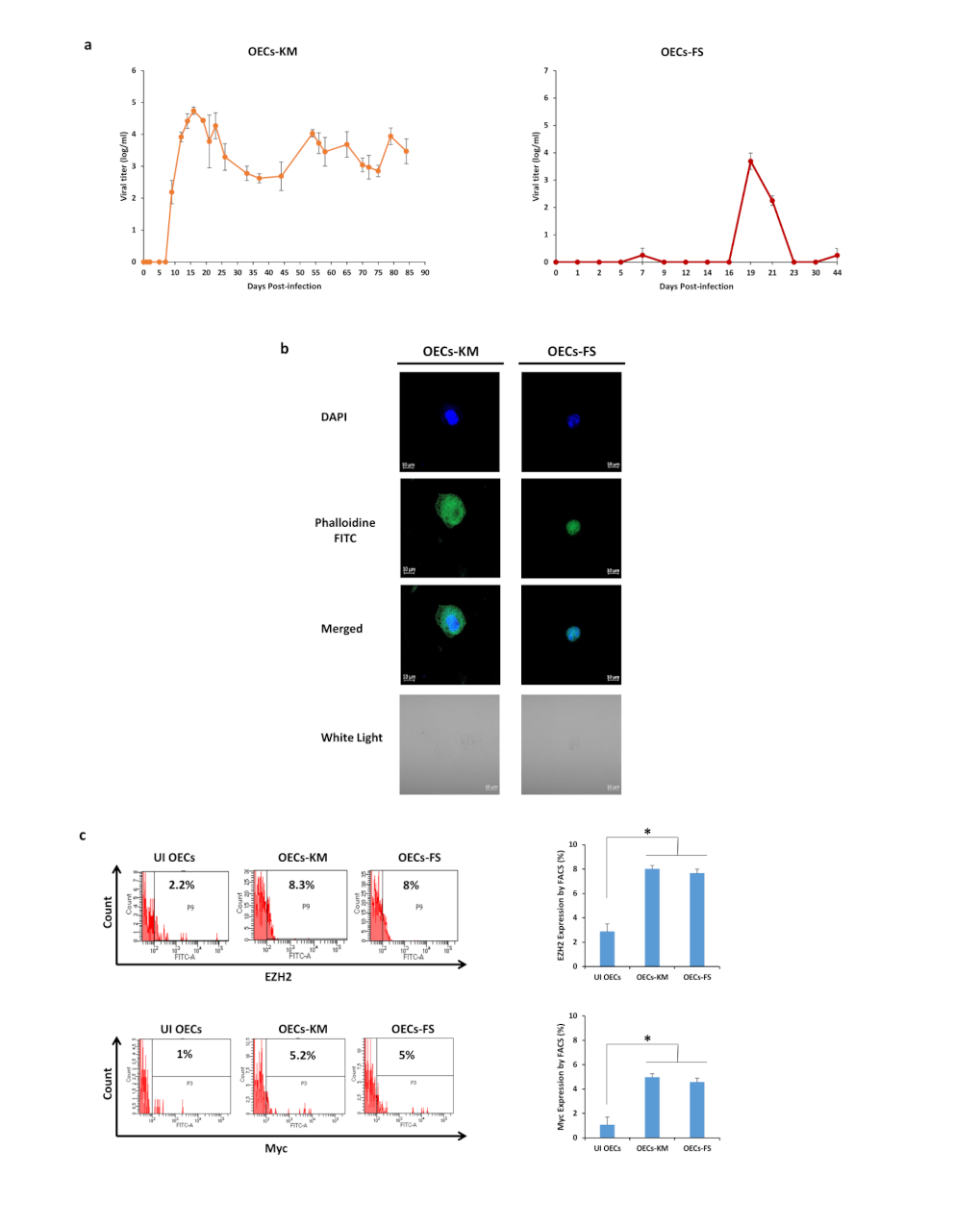


**Supplementary Figure 1. Replication of low risk HCMV strains in OECs cultures. a.** Time-course of the viral titer in the supernatant of OECs infected with HCMV-KM and FS as measured by IE1-qPCR. **b.** Confocal microscopic images of DAPI and phalloidine staining in OECs infected with HCMV-KM and FS. **c.** FACS staining of EZH2 and Myc in uninfected OECs as well as OECs-KM and FS. Data are represented as mean ± SD of two independent experiments. * p-value≤0^.^05.

**
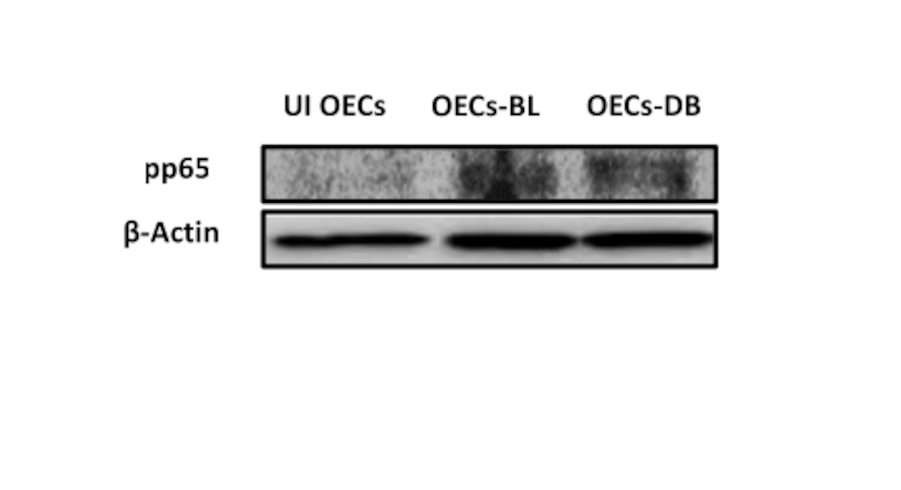
**

**Supplementary Figure 2. Expression of HCMV-pp65 in OECs infected with HCMV-DB and BL.** Immunoblotting data of pp65 in uninfected OECs lysates and OECs infected with HCMV-DB and BL (day 5 post-infection). β-actin was used as loading control.

**
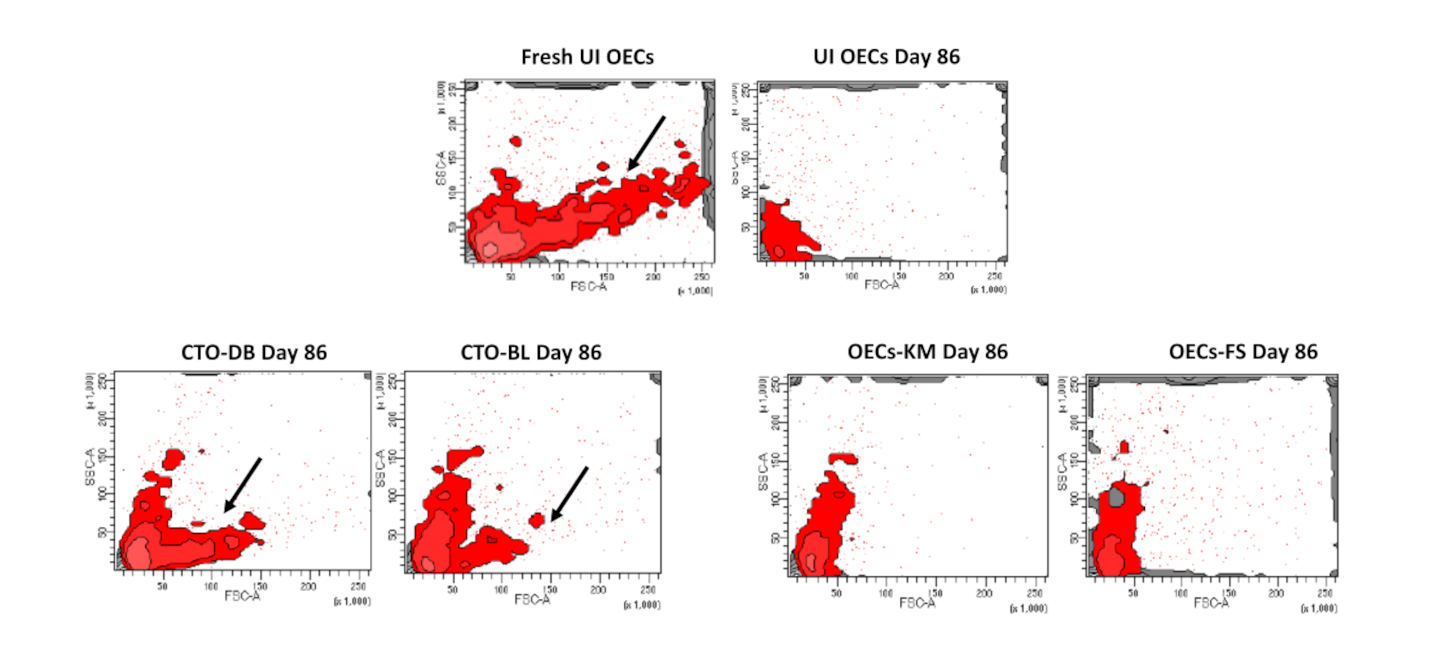
**

**Supplementary Figure 3. Flow cytometric analysis based on FSC and SSC** **of uninfected OECs (fresh and at day 86 post seeding) as well as chronically infected OECs.** Only chronically-infected OECs-DB and BL, namely CTO-DB and CTO-BL cells, and not the chronically-infected OECs-KM and FS were alive at day 86 post-infection (black arrows).

**
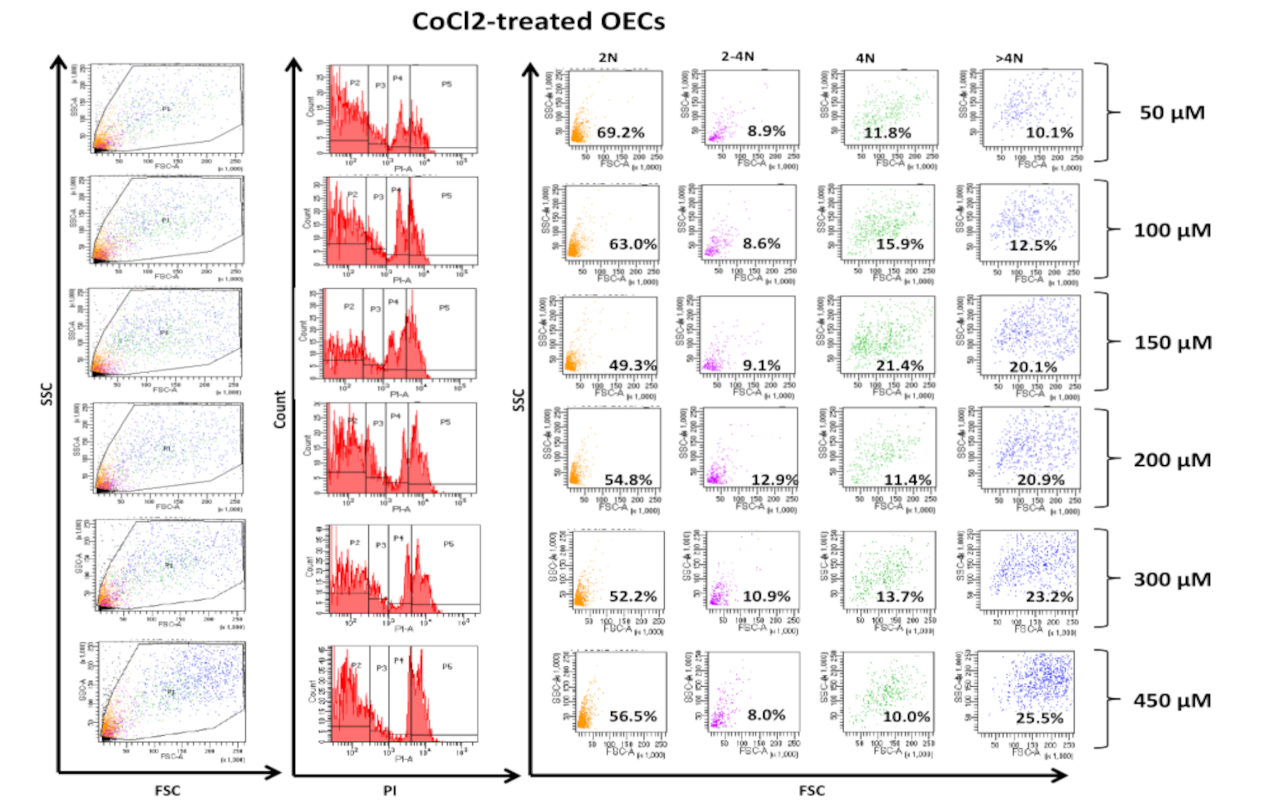
**

**Supplementary Figure 4. PI staining of CoCl2-treated OECs using different drug concentrations (50, 100, 150, 200, 300, and 450** **µM) by FACS.**

**
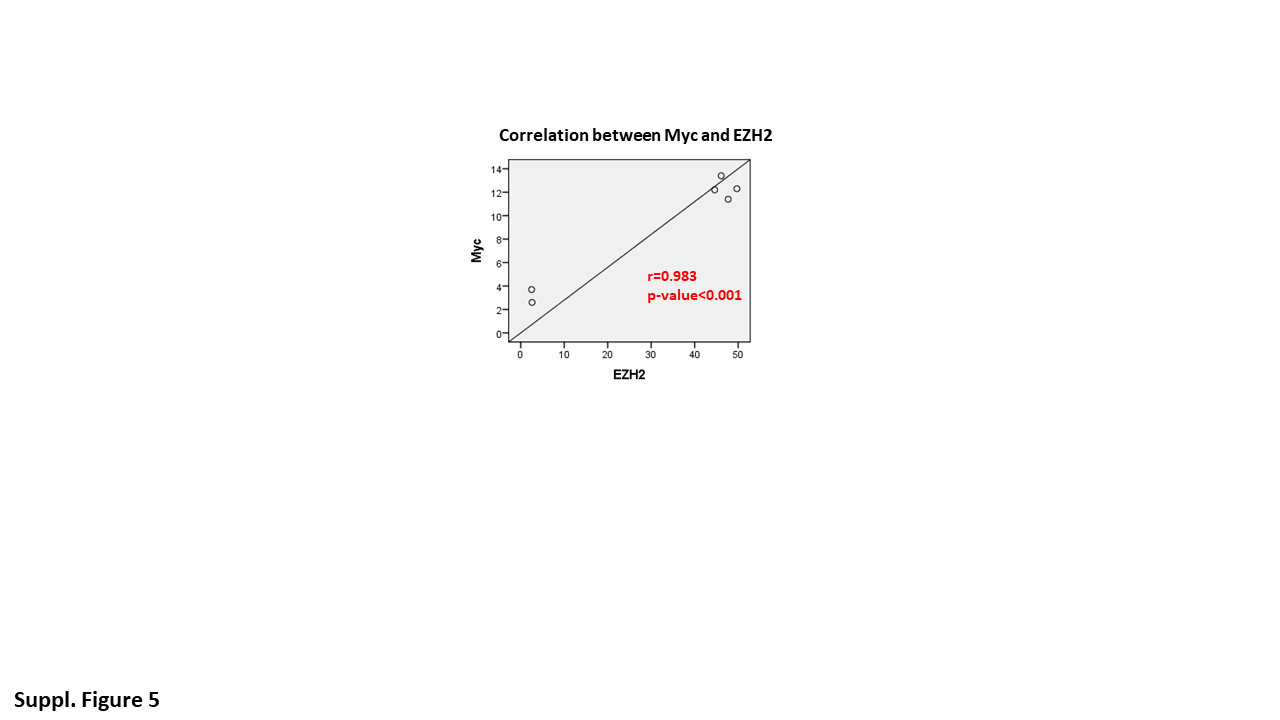
**

**Supplementary Figure 5: The assessment of the correlation between EZH2 and Myc expression in CTO cells.** A significant-positive correlation detected between EZH2 and Myc protein expression in CTO-DB and BL cells. p-values were determined by Pearson’s test.

**
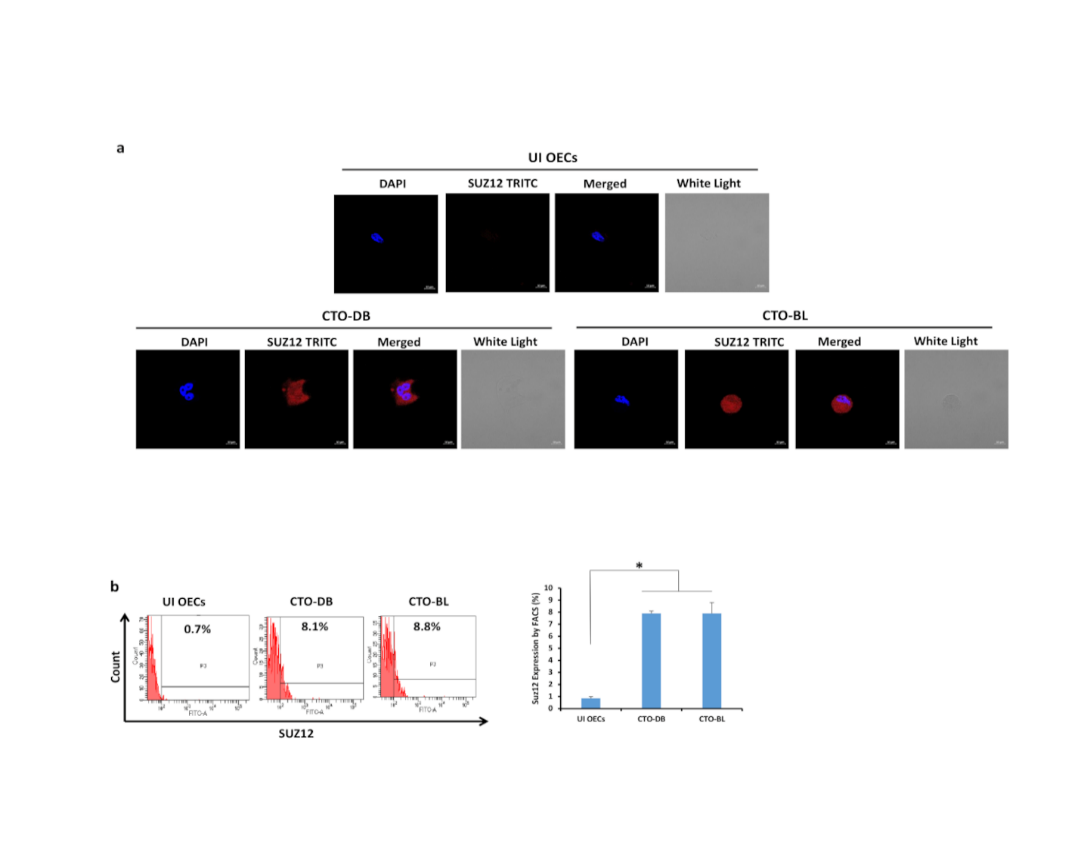
**

**Supplementary Figure 6. Expression of SUZ12 in CTO-DB and BL cells. a,b.** SUZ12 expression by **(a)** confocal microscopy and **(b)** FACS in CTO-DB and BL; uninfected OECs were used as a control. Nuclei were counterstained with DAPI; magnification ×63, scale bar 10 μm. Data are represented as mean ± SD of two independent experiments. * p-value≤0.05.

**
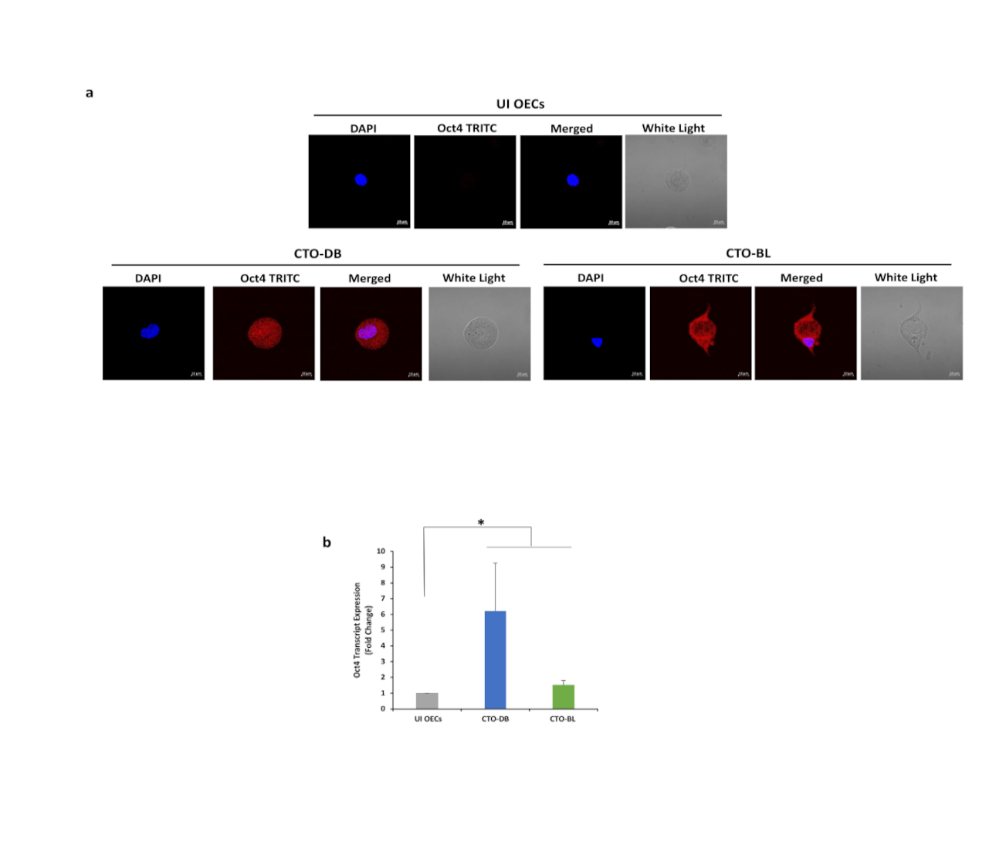
**

**Supplementary Figure 7. Expression of Oct4 in CTO-DB and BL cells. a.** Confocal microscopic images of Oct4 and DAPI staining in CTO-DB and BL cells. UI OECs were used as controls; magnification ×63, scale bar 10 μm. **b.** Oct4 transcript detection by RT-qPCR. Data are represented as mean ± SD of two independent experiments. * p-value≤0.05.

**
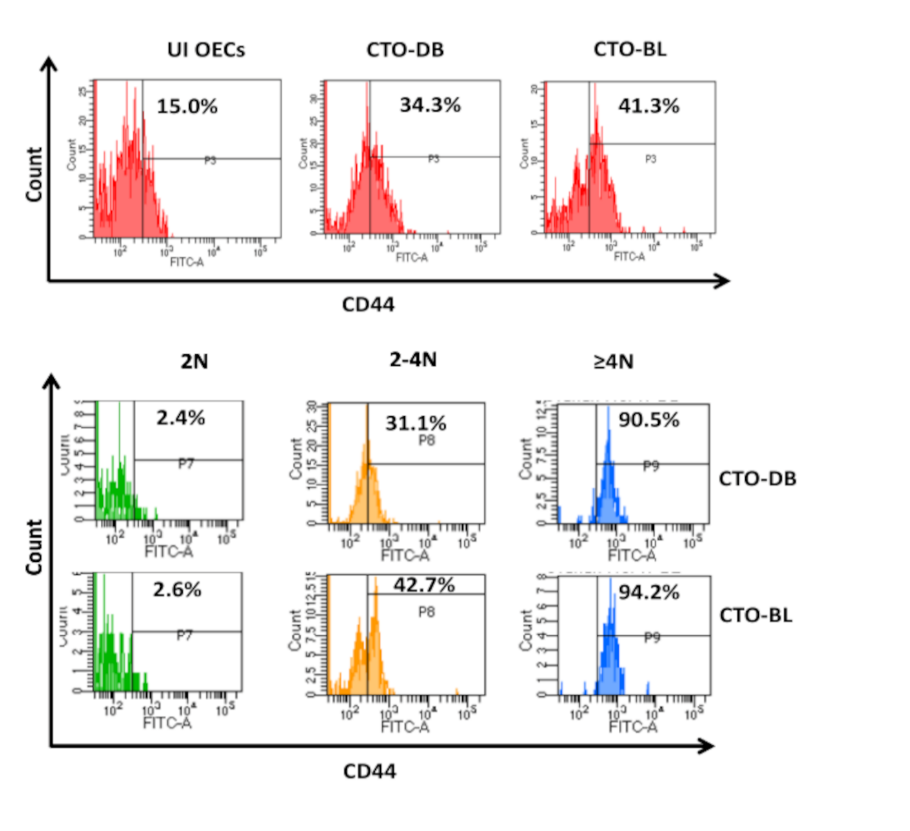
**

**Supplementary Figure 8.** **Expression of CD44 in CTO-DB and BL cells.** FACS staining of CD44 in CTO-DB and BL whole and subpopulations; uninfected OECs were used as a control.

**
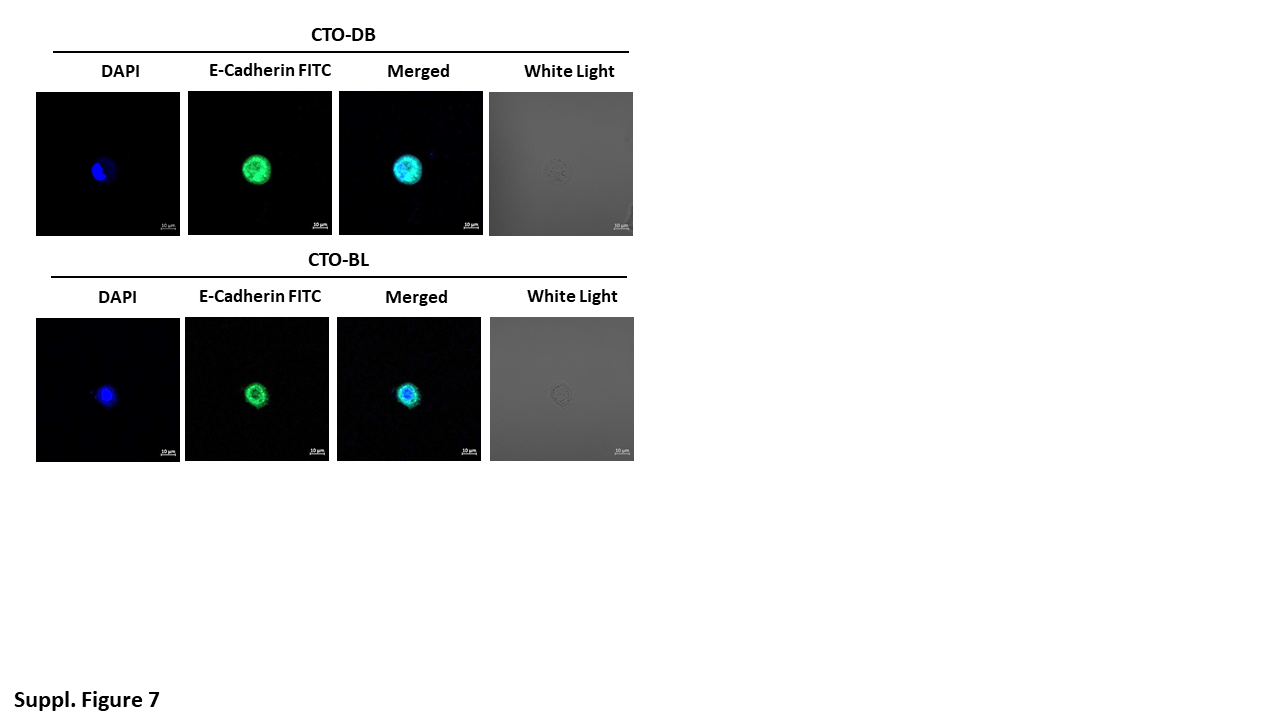
**

**Supplementary Figure 9. Detection of high expression of E-cadherin in several small cells present in CTO-DB and BL cultures.** Confocal microscopic images of E-cadherin and DAPI staining in CTO-DB and BL cultures; magnification ×63, scale bar 10 μm.

**
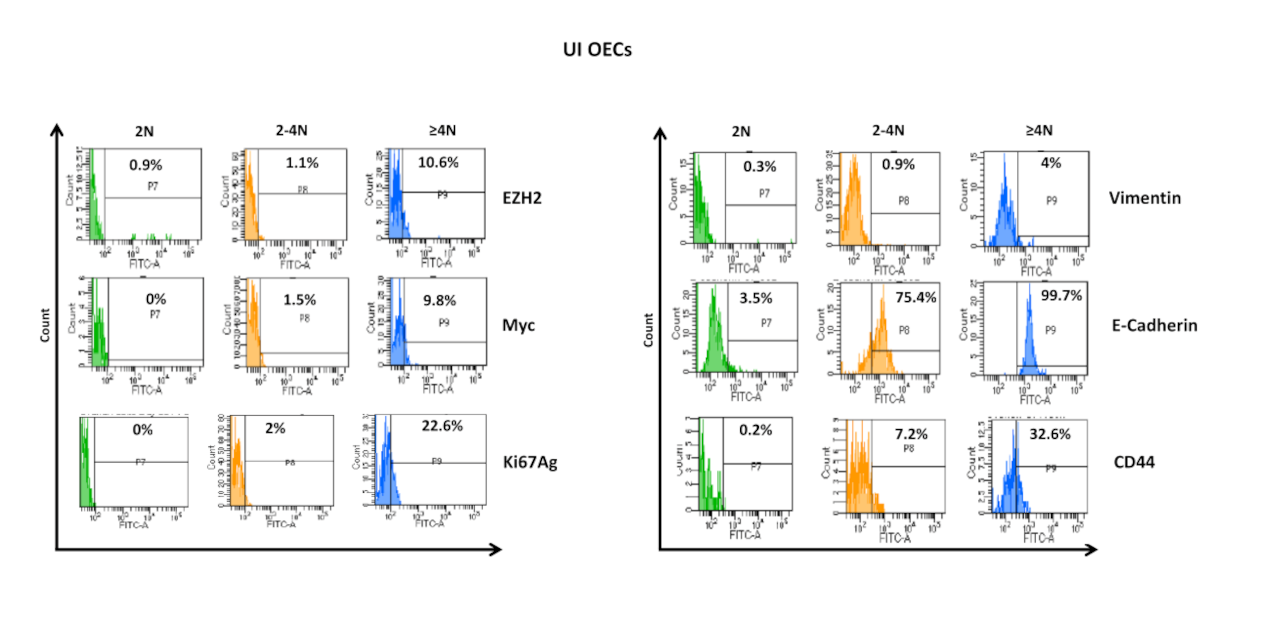
**

**Supplementary Figure 10.** FACS staining of EZH2, Myc, Ki67Ag, Vimentin, E-cadherin, and CD44 in the subpopulations of uninfected OECs.


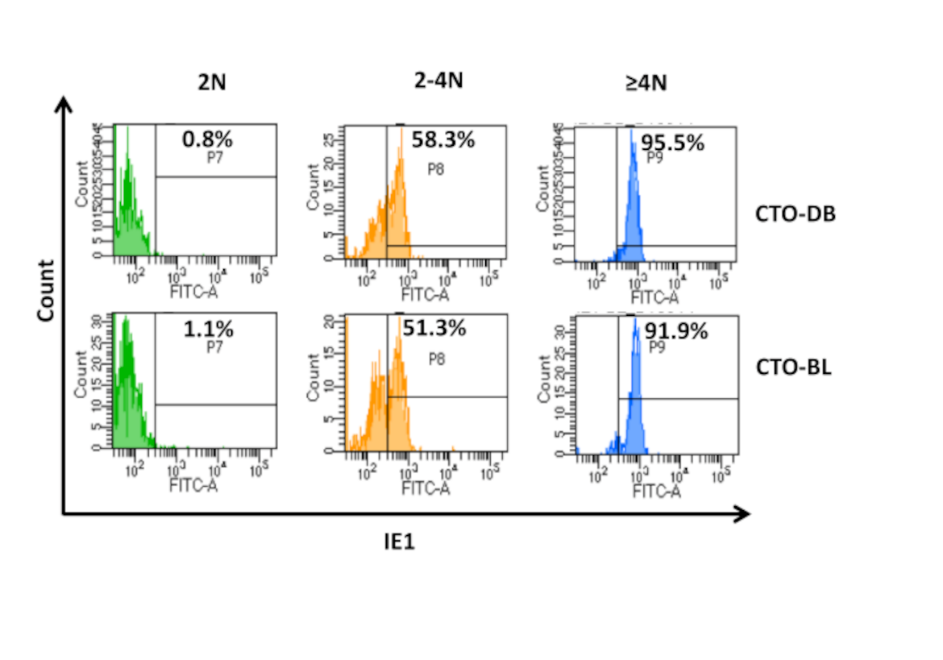


**Supplementary Figure 11. Expression of IE1 in CTO-DB and CTO-BL subpopulations.** FACS staining of IE1 in CTO-DB and BL subpopulations.

**
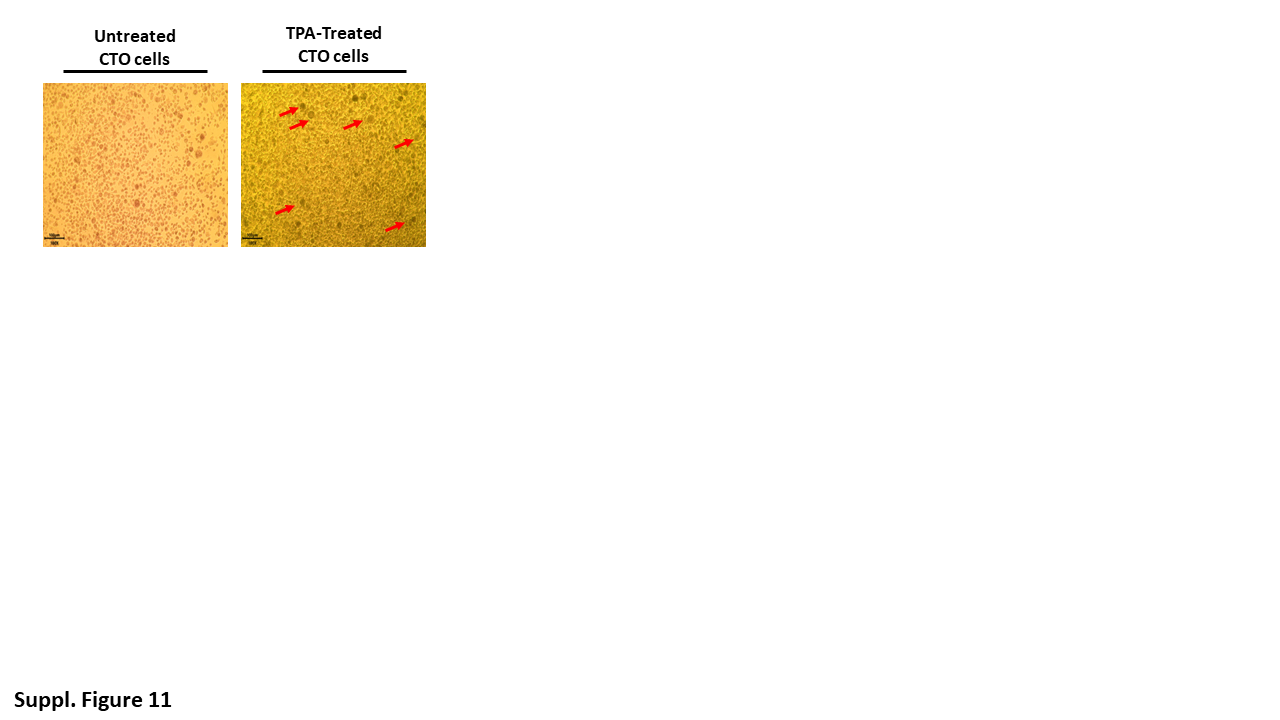
**

**Supplementary Figure 12:** **CTO cultures post-TPA treatment.** Microscopic images of TPA-treated CTO cells; untreated CTO cells were used as a control. Magnification ×100, scale bar 100 μm. Red arrows represent PGCCs found in TPA-treated CTO cultures.


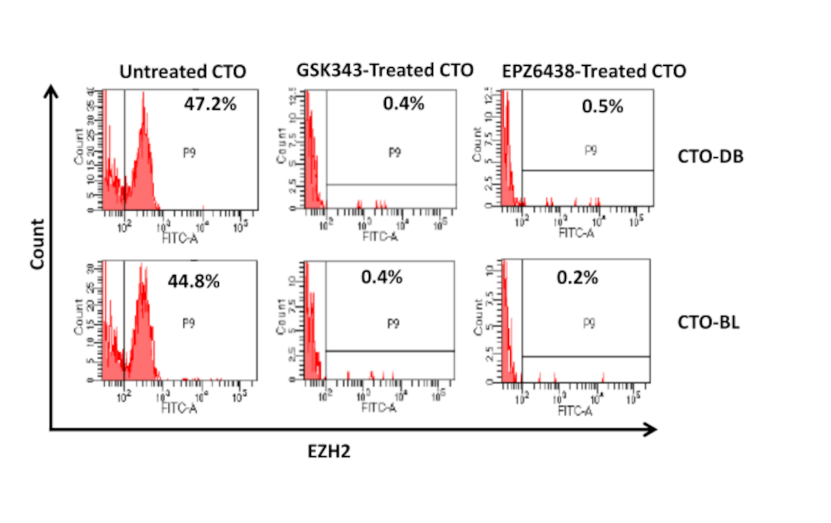


**Supplementary Figure 13.** EZH2 expression in untreated CTO-DB/BL and CTO-DB/BL treated with 0.1 µM of GSK343 and EPZ6438 by FACS.

**
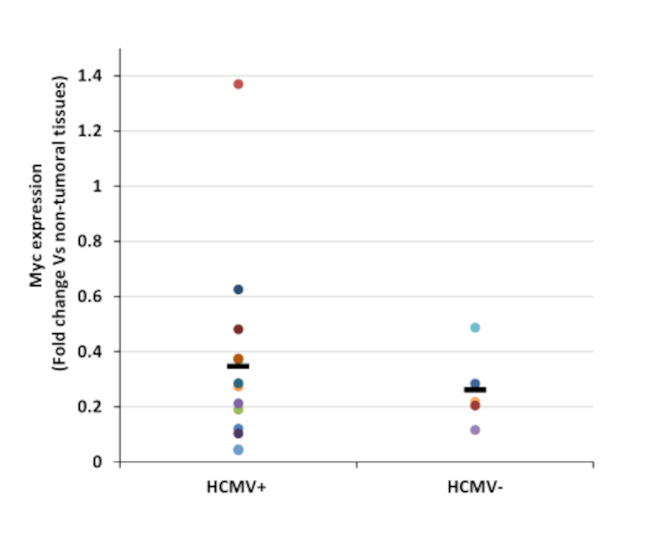
**

**Supplementary Figure 14. Myc Expression in ovarian cancer biopsies.** Scattered plot representing Myc expression in HCMV-positive and negative ovarian tumor biopsies by RT-qPCR.


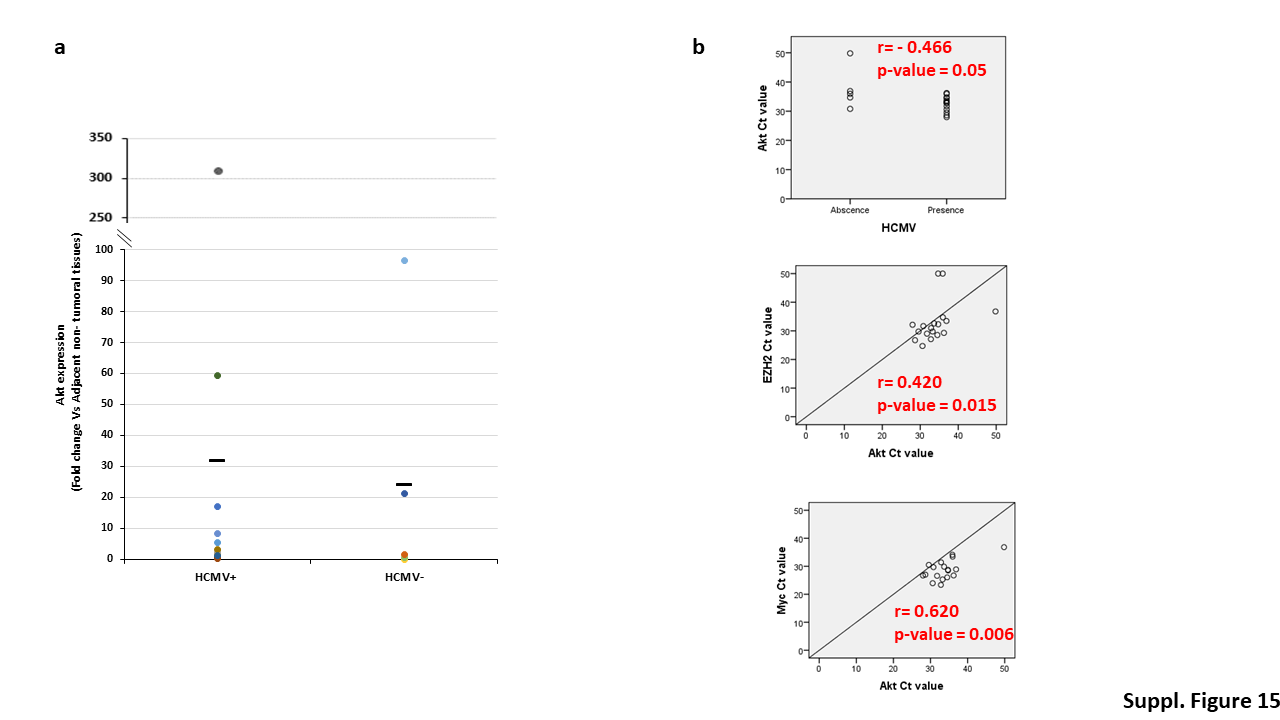


**Supplementary Figure 15. Akt Expression in ovarian cancer biopsies. a.** Scattered plot representing Akt expression in HCMV-positive and negative ovarian tumor biopsies by RT-qPCR. **b.** A correlation detected between Akt expression (Ct value) and HCMV presence, in addition to Akt expression and EZH2 as well as Myc expression. p-values were determined by Spearman, Kendall's Tau, and Pearson correlation tests, respectively.

# Supplementary Tables

**Supplementary Table 1. List of primers used.**

| **Primer** | **Primer Sequence** |
| --- | --- |
| IE1-forward | 5'-CGACGTTCCTGCAGACTATG-3' |
| IE1-reverse | 5'-TCCTCGGTCACTTGTTCAAA-3' |
| UL69-forward | 5’-GGGATGTCGATGACTCCCTTC-3’ |
| UL69-reverse | 5’-GTCGCTATTGGATCTCACCGT-3’ |
| EZH2-forward | 5’-TCGTGCCCTTGTGTGATAGC-3’ |
| EZH2-reverse | 5’-TCTCGGACAGCCAGGTAGC-3’ |
| MYC-forward | 5’-ACACCCTTCTCCCTTCG-3’ |
| MYC-reverse | 5’CCGCTCCACATACAGTCC3’ |
| SOX2-forward | 5′-GGGAAATGGAGG GGTGCAAAAGAGG-3′ |
| SOX2-reverse | 5′-TTGCGTGAGTGT GGATGG GATTGGTG-3′ |
| Nanog-forward | 5′-TCCTCCTCTTCCTCTATACTAAC-3′ |
| Nanog-reverse | 5′-CCC ACAATCACAGGCATAG-3′ |
| Oct4-forward | 5′-TGGAGAAGGAGAAGCTGGAGCAAAA-3′ |
| Oct4-reverse | 5′-GGCAGAGGTCGTTTGGCTGAATAGACC-3′ |
| Akt-forward | 5’-ATCCCCTCAACAACTTCTCAGT-3’ |
| Akt-reverse | 5’-CTTCCGTCCACTCTTCTCTTTC-3’ |
| β-2-Microglobulin-forward | 5’-GATGAGTATGCCTGCCGTGTG-3’ |
| β-2-Microglobulin-reverse | 5’-CAATCCAAATGCGGCATCT-3’ |

**Supplementary Table 2. List of antibodies used.**

| **Antibody** | **Catalog Number/Source** |
| --- | --- |
| Anti-Myc Tag | 06-549-25UG/Merck KGaA, (Darmstadt, Germany) |
| EZH2 | AB_2793397/Active Motif (Carlsbad, CA, USA) |
| Ki67Ag | BD-556026/BD Biosciences (Franklin Lakes, USA) |
| CMV pp72 (IE1) | SC-69834/Santa Cruz Biotechnology (CA, USA) |
| IE1 | ab53495/Abcam (Cambridge, UK) |
| Oct4 | ab19857/Abcam (Cambridge, UK) |
| SOX2 | ab97959/Abcam (Cambridge, UK) |
| pp65 | SC-52401/Santa Cruz Biotechnology (CA, USA) |
| CD44 | BD-555478/BD Biosciences (Franklin Lakes, USA) |
| Vimentin | SC-6260/Santa Cruz Biotechnology (CA, USA) |
| E-cadherin | SC-8426/Santa Cruz Biotechnology (CA, USA) |
| p53 | SC-47698/Santa Cruz Biotechnology (CA, USA) |
| Rb | SC-102/Santa Cruz Biotechnology (CA, USA) |
| pRb | SC-377528/Santa Cruz Biotechnology (CA, USA) |
| Phalloidine | ab235137/ Abcam (Cambridge, UK) |
| Nanog | SC-293121/Santa Cruz Biotechnology (CA, USA) |
| SUZ12 | AB_2614929/Active Motif (Carlsbad, CA, USA) |
| FITC-conjugated anti-mouse antibody | BD- 553399/BD Biosciences (Franklin Lakes, USA) |
| PE-conjugated anti-mouse antibody | BD-551436/BD Biosciences (Franklin Lakes, USA) |
| FITC-conjugated anti-rabbit antibody | ab6717/Abcam (Cambridge, UK) |
| FITC-conjugated Goat Anti-Mouse | BD-555988/BD Biosciences (Franklin Lakes, USA) |
| FITC-conjugated Rat Anti-Mouse | BD-553443/BD Biosciences (Franklin Lakes, USA) |
| FITC Mouse IgG2a, κ Isotype Control | BD-553456/BD Biosciences (Franklin Lakes, USA) |
| Propidium Iodide | P3566/Life Technologies (Eugene, USA) |

**Supplementary Table 3. Clinical data and treatments of the OC patients.**

| **Biopsy #** | **Age** | **Tumor** | **Metastasis** | **Chemotherapy** |
| --- | --- | --- | --- | --- |
| 1 | 67 | HGSOC | + | carboplatin – gemcitabine - bevacizumab |
| 2 | 64 | HGSOC | + | carboplatin -paclitaxel |
| 3 | 50 | HGSOC | + | bevacizumab-carboplatin -paclitaxel -pegylated liposomal doxorubicin-HIPEC |
| 4 | 82 | HGSOC | + | carboplatin – gemcitabine – bevacizumab - paclitaxel |
| 5 | 63 | HGSOC | + | carboplatin -paclitaxel - pegylated liposomal doxorubicin |
| 6 | 48 | HGSOC | + | bevacizumab – carboplatin – paclitaxel - pegylated liposomal doxorubicin |
| 7 | 57 | HGSOC | + | topotecan |
| 8 | 79 | HGSOC | - | carboplatin -paclitaxel |
| 9 | 70 | HGSOC | + | carboplatin – gemcitabine - bevacizumab |
| 10 | 57 | HGSOC | - | carboplatin - cyclophosphamide |
| 11 | 70 | HGSOC | + | carboplatin -paclitaxel - pegylated liposomal doxorubicin – gemcitabine -topotecan |
| 12 | 54 | HGSOC | + | carboplatin -paclitaxel - bevacizumab |
| 13 | 56 | HGSOC | + | carboplatin -paclitaxel |
| 14 | 88 | HGSOC | + | carboplatin -paclitaxel |
| 15 | 60 | HGSOC | + | carboplatin -paclitaxel |
| 16 | 64 | HGSOC | + | carboplatin -paclitaxel |
| 17 | 57 | HGSOC | + | docetaxel - carboplatin -paclitaxel |
| 18 | 44 | HGSOC | + | carboplatin - paclitaxel |

Metastasis: +: Presence / -: Absence

HIPEC: hyperthermic intraperitoneal chemotherapy
